# Real-Time Multiscale Monitoring and Tailoring of Graphene Growth on Liquid Copper

**DOI:** 10.1021/acsnano.0c10377

**Published:** 2021-06-01

**Authors:** Maciej Jankowski, Mehdi Saedi, Francesco La Porta, Anastasios C. Manikas, Christos Tsakonas, Juan S. Cingolani, Mie Andersen, Marc de Voogd, Gertjan J. C. van Baarle, Karsten Reuter, Costas Galiotis, Gilles Renaud, Oleg V. Konovalov, Irene M. N. Groot

**Affiliations:** † Université Grenoble Alpes, CEA, IRIG/MEM/NRS, 38000 Grenoble, France; ‡ Leiden Institute of Chemistry, Leiden University, P.O. Box 9502, 2300 RA Leiden, The Netherlands; § ESRF-The European Synchrotron, 71 Avenue des Martyrs, 38043 Grenoble, France; ∥ FORTH/ICE-HT and Department of Chemical Engineering, University of Patras, Patras 26504, Greece; ⊥ Chair for Theoretical Chemistry and Catalysis Research Center, Technische Universität München, Lichtenbergstrasse 4, 85747 Garching, Germany; # Leiden Probe Microscopy (LPM), Kenauweg 21, 2331 BA Leiden, The Netherlands

**Keywords:** CVD graphene, liquid metal catalyst, two-dimensional materials, Raman spectroscopy, X-ray diffraction, radiation optical microscopy, self-organization

## Abstract

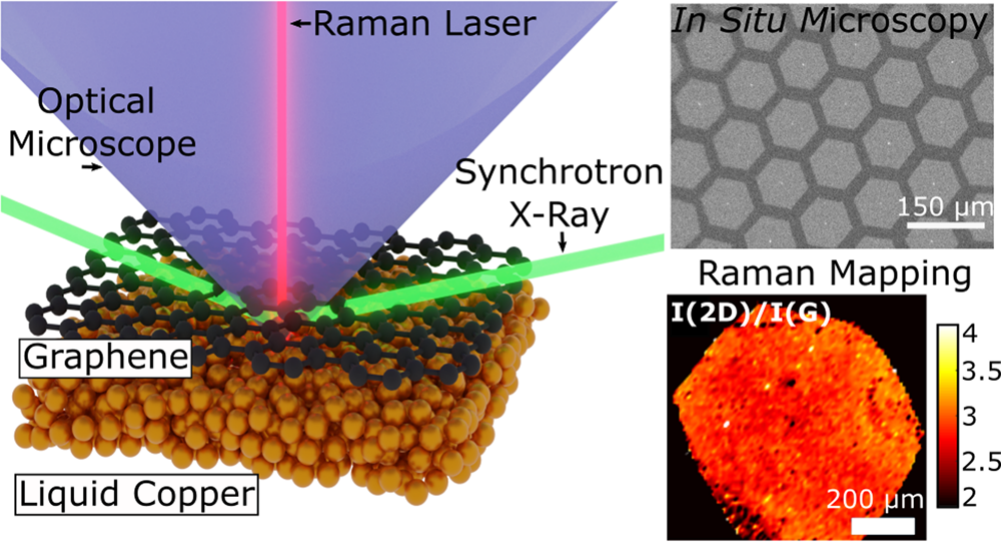

The synthesis of large, defect-free two-dimensional materials (2DMs) such as graphene is a major challenge toward industrial applications. Chemical vapor deposition (CVD) on liquid metal catalysts (LMCats) is a recently developed process for the fast synthesis of high-quality single crystals of 2DMs. However, up to now, the lack of *in situ* techniques enabling direct feedback on the growth has limited our understanding of the process dynamics and primarily led to empirical growth recipes. Thus, an *in situ* multiscale monitoring of the 2DMs structure, coupled with a real-time control of the growth parameters, is necessary for efficient synthesis. Here we report real-time monitoring of graphene growth on liquid copper (at 1370 K under atmospheric pressure CVD conditions) *via* four complementary *in situ* methods: synchrotron X-ray diffraction and reflectivity, Raman spectroscopy, and radiation-mode optical microscopy. This has allowed us to control graphene growth parameters such as shape, dispersion, and the hexagonal supra-organization with very high accuracy. Furthermore, the switch from continuous polycrystalline film to the growth of millimeter-sized defect-free single crystals could also be accomplished. The presented results have far-reaching consequences for studying and tailoring 2D material formation processes on LMCats under CVD growth conditions. Finally, the experimental observations are supported by multiscale modeling that has thrown light into the underlying mechanisms of graphene growth.

## Introduction

Reproducible mass production of large, defect-free two-dimensional materials (2DMs) such as graphene is a major challenge toward their industrial applications. Chemical vapor deposition (CVD) is to date the most promising method to produce large, high-quality graphene sheets.^1,2^ CVD involves decomposing a gas precursor on a hot catalyst and its subsequent diffusion, followed by flake nucleation, growth, and coalescence into a continuous 2D layer. Graphene is often grown using a CH_4_ precursor on a solid copper catalyst at ∼1270 K.^2^ High nucleation rate and growth at random substrate positions result in a polycrystalline layer, whose properties are affected by the purity, roughness, crystallographic structure, and domain boundaries of the substrate.^3,4^ Notable achievements for graphene growth on solid copper have been reported, such as the stitching of aligned graphene domains into a single-crystal film^5^ or the growth of inch-size single crystals from one nucleus.^6,7^ However, these results were obtained using conditions that are hard to implement at an industrial scale. Moreover, because of the thermal expansion mismatch between graphene and copper, high residual stresses develop upon cooling to ambient temperature, inducing wrinkling and folding of the final graphene film.^8^ Finally, after cooling to room temperature, special chemical methods (*e.g*., etching the catalyst away using an acid) are applied to separate and transfer the graphene due to the presence of considerable van der Waals forces between the graphene and solid substrate. This leads to further contamination and damage to graphene.^9^

Recently, liquid metal catalysts (LMCats), *e.g*., molten copper, have been employed for the fast growth of uniform hexagonal graphene and other 2DMs flakes and films of significantly higher quality, while using experimental temperature, pressure, and flow conditions that are comparable to those used with solid catalysts.^10−12^ One of the main advantages of LMCats over solid substrates is their surface structure. LMCats consist of an atomically flat isotropic melt, with low surface roughness and absence of defects characteristic for solid substrates, such as atomic terraces, dislocations, and impurities, that impact graphene growth.^13^ Moreover, the crystalline solid surface imposes preferential orientation, the formation of moiré superlattices, and induces strain in the grown graphene.^14^ The absence of a crystalline structure on LMCats allows the formation of graphene crystals in any in-plane orientation, freely moving and rotating during the growth and without substrate-induced strain. This leads to much easier growth of high-quality graphene crystals, their self-assembling, and the formation of uniform layers.^15−17^ Compared with solids, the measured nucleation density is at least 1 order of magnitude lower,^18^ leading to fewer domain boundaries and bigger domains. Also, the growth speed is much higher,^18^ which is very beneficial in producing large-area flakes, caused by high diffusion rates,^18^ and catalytic activity of LMCats.^19^ The weak interactions between LMCats and the grown layers suggest the possibility to attempt for a direct transfer without LMCat solidification and complex postgrowth transfer processes. Such a challenging process is both theoretically possible (see Supplementary Note 1), and practically achievable, as first successful trials have already been reported.^20^ The main focus in the investigation of graphene on LMCats is concentrated on the graphene–copper system and to a less extent on other liquid metals. However, successful growth examples on LMCats other than liquid copper,^21^*e.g*., PtSi,^22^ Ni,^23^ Ga,^24^ CuSn, and Sn,^25^ reveal the existence of a broader family of LMCats, allowing the growth of graphene at significantly lower temperatures^24^ than currently used in the standard CVD growth on solid copper (1100–1300 K).^26^ Moreover, the reports on the successful synthesis of *h*-BN,^27^ MoC,^28^*h*-BN-graphene heterostructures,^27^ GaN,^29^ thin oxides,^30^ and others^21^ show a broader range of LMCats applications allowing fast production of high-quality 2D materials beyond graphene.

Several examples of graphene growth on LMCats with different morphologies have been reported,^12^ and intense efforts have been made to optimize the CVD process. In general, even in the case of solid catalysts, there are limited reports on real-time observation of graphene growth, with notable examples of those employing radiation-mode optical microscopy,^31^ environmental scanning electron microscopy,^32^ or *in situ* reflectance spectroscopy.^33^ However, on liquid catalysts, and contrary to the ultrahigh vacuum^34^ and low-pressure^32^ CVD on solid substrates, the lack of precise, multiscale *in situ* techniques enabling direct feedback on the growth parameters of atmospheric pressure CVD (including temperature, gas composition, and pressures), has led primarily to empirical recipes. Such recipes intrinsically suffer from a limited understanding of the graphene formation process and low reproducibility of the product due to the complex and stochastic nature of the growth phenomena. For the realization of a stable, continuous 2DM production process, *in situ* multiscale monitoring of defects and morphologies from atomic to macroscopic scales and real-time feedback control on process parameters is mandatory as an additional level of complexity is added by the continuous movement, rotation, and mutual interactions of graphene crystals on molten metal. However, until now, there were significant hurdles against the realization of *in situ* monitoring techniques for 2DM growth on LMCats, including heat and evaporation of the liquid metal, intense thermal radiation, curved and dynamic nature of the liquid surface, and the presence of reactive CVD gas at close to atmospheric pressure.

Here we report the successful implementation of four *in situ* techniques for multiscale monitoring of graphene growth on liquid copper at 1370 K and under atmospheric pressure CVD conditions (see Methods and Supplementary Note 2). Radiation-mode optical microscopy, which has been previously demonstrated during graphene growth on solid copper,^31^ provides essential information on growth morphology and dynamics in real time at the macroscopic scales. We find (see Supplementary Note 3) that radiation-mode optical microscopy is extremely sensitive to the thickness of the grown graphene on liquid copper and allows us to demonstrate the growth of single-layer graphene (SLG) or to detect any part that is multilayer.^31^ Raman spectroscopy, which has been employed *in situ* (at high temperatures), confirms monolayer graphene’s presence and yields information about its crystallinity and defects from mesoscopic to nanoscales. At the atomic scale, the lattice constant and corrugation of graphene sheets floating on liquid copper are derived from Bragg rods’ position and angular spread measured by synchrotron grazing incidence X-ray diffraction (GIXD). The number of graphene layers, roughness, and the separation between graphene and liquid copper are provided by synchrotron X-ray reflectivity (XRR). Real-time monitoring allows us to tailor the crystal size, shape, and quality while optimizing the growth speeds. This achievement should enable 2DMs’ applications in domains for which reproducible specifications are of paramount importance, *e.g*., microelectronic and photonic industries.^35^

To demonstrate the wealth of information and control capability that can be achieved by multiscale *in situ* monitoring, we first show the results of CVD growth processes for which the nucleation of graphene seeds is induced by an injection of a short pulse of methane at high partial pressure. This procedure produces many flakes that grow and form a superordered assembly due to short- and long-range interactions, as explained by multiscale simulations. Ultimately, they merge into a continuous film; however, slight misorientations of neighboring flakes remain upon their coalescence, ultimately leaving domain boundaries where they have merged. We next use our monitoring and feedback-control possibility to improve the flakes’ ordering and reduce the remnant defects upon merging. Although this procedure decreases the defect density, perfect stitching of neighboring flakes could not be achieved, as revealed by etching under higher hydrogen partial pressure. Finally, we tailor the growth parameters to nucleate only a single flake and grow it to millimeter size. The obtained spectra using X-ray scattering and Raman spectroscopy compare well to those of single-layer exfoliated graphene.

## Results and Discussion

### Growth of Graphene on Liquid Copper by “Gas-Pulse” Injection

**Figure 1:**
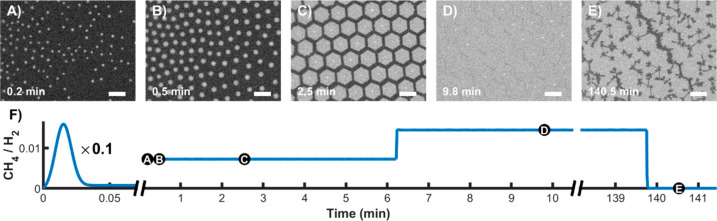
“Pulse” growth of graphene on liquid copper (see also Movie 1) and its characterization. Graphene growth on liquid copper from multiple seeds. Graphene growth (A)–(D) and subsequent etching (E) recorded by *in situ* optical microscopy. See Movie 1. The length of the scale bars corresponds to 100 μm. (F) *p*(CH_4_)/*p*(H_2_) evolution for the (A)–(E) growth sequence.

Figure 1A–E and Movie 1 show real-time optical microscopy measurements recorded at different stages of a typical “pulse” CVD graphene growth on liquid copper. The “pulse” (applied between 0 and 10 s of Movie 1) refers to a sudden release of a high concentration of methane gas (Figure 1F) into the main gas stream of the argon/hydrogen mixture, which triggers a high graphene nucleation rate. Subsequently, a continuous flow of low-concentration methane (pressure ratio *p*(CH_4_)/*p*(H_2_) = 1/138) is maintained, leading to the gradual growth of the nucleated flakes (starting at 10 s of Movie 1). These flakes are seen as white features appearing on the surface, as the area covered by graphene has a slightly higher emissivity than liquid copper at this temperature. For almost all flakes, a central seed consisting of a needle-like 3D graphitic structure is visible. These structures are etched in the final stages of the growth,^36^ which is self-limiting to the SLG (see Supplementary Note 4). After a few more tens of seconds, the flakes increase in size and move closer to each other, indicating the presence of an attractive long-range interaction between them. Strikingly, although the flakes grow in size, they do not merge but remain separated by a gap, which is evidence of the presence of a short-range repulsive interaction between them. They next self-assemble in a near-perfect hexagonal network with a separation that converges to a constant gap value of 20–40 μm (Figure 1C and Supplementary Figure 4) while the flakes continue to grow. In the final stage of the growth (after ∼3 min 30 s), the aligned flakes start to merge. Our general observation is that the closure of the gaps coincides with the moment that the assembly of flakes grows large enough to reach the borders of the liquid copper pool. At this point, the ordering is perturbed, and only local order is preserved. The gap between the flakes slowly disappears with growth time, leading to continuous SLG (see below). The SLG nature of the flakes is unambiguously deduced from the contrast in radiation-mode optical microscopy. We have consistently observed that adjacent flakes avoid any overlapping even when the gap between them vanishes and the layer closes. This is not surprising as the adhesion energy between two overlapping graphene layers is less than between graphene and liquid copper (0.23 and 0.30 [J m^–2^], respectively).^37,38^ Also, once the edges of the adjacent coalescing flakes reach each other, the formation of covalent bonds between their unsaturated edge carbon atoms is energetically more favorable than the weak van der Waals interaction between hypothetically overlapping flakes. Such overlapping events are energetically unfavorable for free-floating flakes.^39^Figure 1D shows the layer just before complete closure. Increasing (actually doubling to speed up the process) the H_2_ concentration allows for etching of the layer, primarily attacking the defect sites, including domain boundaries (Figure 1E). The etching allows revealing the domain boundaries and point defects present in the grown layer. The linear cavities are formed around domain boundaries,^40^ whereas compact cavities appear around point defects^41^ (see Supplementary Note 5).

### Driving Force for Self-Assembly

**Figure 2:**
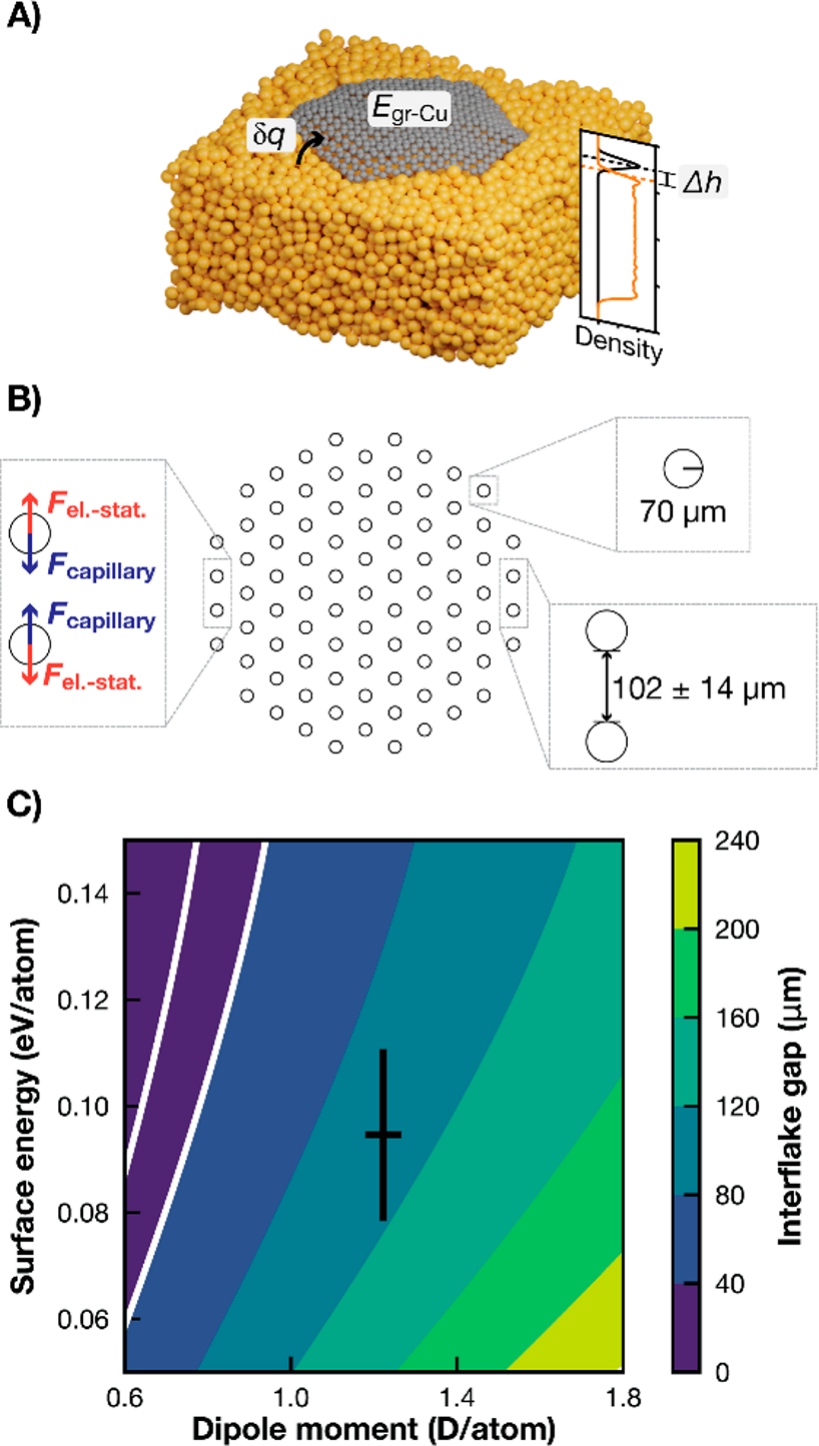
Theoretical modeling of graphene flake interactions on liquid copper. (A) Snapshot of an MD simulation of a hexagonal graphene flake on a liquid Cu slab. Calculated average properties, namely, the charge transfer from the substrate to the flake, δ*q*, the interaction energy between graphene and copper, *E*_Gr–Cu_, the height of the flake above the surface, Δ*h*, as well as the accompanying density profile, are indicated. The orange line corresponds to the density of Cu atoms within the hexagon’s inscribed circle and the black line to the C atomic density. The densities have been arbitrarily scaled for better visualization. (B) Schematic representation of the group of interacting flakes used to calculate the separation distance. The zoom-ins indicate the two governing forces, the used flake radius, and the optimized distance. (C) Average distance between flakes as predicted by the capillary-electrostatic model as a function of the two model parameters. The black cross corresponds to the dipole moment and interaction energy obtained from our MD simulations with their corresponding standard deviations. The white lines denote 20 and 40 μm gap distances (area in agreement with the experiments).

Despite many variations of the growth conditions (see Movies 1 and 2), domain boundaries are always present in the final continuous SLG, which is always polycrystalline. These boundaries are due to a fluctuation of the azimuthal orientation of adjacent domains at the late coalescence stage, and they contain a higher density of defects and impurities than the domain interior. This is surprising for a liquid substrate, where the lack of pinning sites and preferred orientation was expected to lead to continuous, domain-boundary-free graphene. To date, a theoretical model for the self-assembly and persistent interflake separation is still lacking. From gravitational interactions^15^ to the shape of the electrostatic potential around the flakes^42^ to the flow of gases in the reactor,^43^ various driving forces have been proposed, but none of these studies have provided a convincing theory able to bridge the microscopic to mesoscopic length scales involved. Here, we employ a multiscale modeling approach; that is, we carry out molecular dynamics (MD) simulations of hexagonal graphene flakes on a liquid Cu surface (see Figure 2A) to derive input parameters for a mesoscopic theory based on short-range repulsive electrostatic and long-range attractive capillary interactions. The latter only manifests itself on liquid surfaces. Interactions between particles on a fluid–fluid interface have been previously rationalized in terms of the deformation of the fluid surface around the particles as originating from the three-phase contact angle.^44^ The characteristic length scale for such interactions is the capillary length, for which we calculate a value of 4 mm for Cu at 1370 K; see Supplementary Note 6. For spherical particles and particle sizes much smaller than the capillary length, analytical expressions for the attractive force between capillary monopoles have been derived by Danov and Kralchevsky.^45^ Apart from the capillary length, they only depend on the small contact angle between the meniscus and the circular contact line of the particle. Using Young’s equation, this contact angle can, in turn, be related to the interfacial energy between the flake and the Cu surface, *E*_Gr–Cu_, for which our MD simulations extrapolated to the large-flake limit give a value of 95 ± 16 meV per C; see Supplementary Note 6.

At the same time, the variable-charge COMB3 potential^46^ employed in the MD simulations predict a charge transfer, δ*q*, from the liquid Cu surface to the electronegative flakes of 0.0445 ± 0.0001 electrons per C atom in the large-flake limit. At the metallic surface, this doping of the graphene flake, resulting from the equilibration of the Fermi levels,^47^ is accompanied by the buildup of an image charge in the conductor. We verify by finite-element calculations (see Supplementary Note 5) that the charge distribution inside a mesoscopic hexagonal flake is essentially homogeneous, allowing us to approximate the repulsive electrostatic forces as simple dipole interactions. Making use of the calculated flake–substrate distance, Δ*h*, of 2.86 ± 0.10 Å, we arrive at a dipole moment of 1.22 ± 0.04 D per C atom. We define the flake–substrate distance as the distance between the graphene layer center and the liquid copper surface inflection point on the gas-copper electron density profile. As shown in Figure 2B, we apply our model to a self-aligned ensemble of 85 particles with the superposition of the longer-ranged attractive capillary attractions and find the optimum interflake distance to be 102 ± 14 μm. In Figure 2C, the dependence of this predicted distance on the model parameters is presented. We ascribe the remaining quantitative difference to the experimentally observed interflake distance of ∼40 μm to the simplicity of the model employed here. Apart from inadequacies of the MD simulations (approximate interatomic potential, the finite size of the simulation cell), we expect in particular higher-order capillary interactions arising for nonspherical particles^45^ and presently not considered electrocapillary interactions^45,48^ to provide an additional attraction that would further shrink the optimum interflake distance.

### Structural Characterization: *In Situ* Raman Spectroscopy, Synchrotron X-ray Diffraction, and Reflectivity

**Figure 3:**
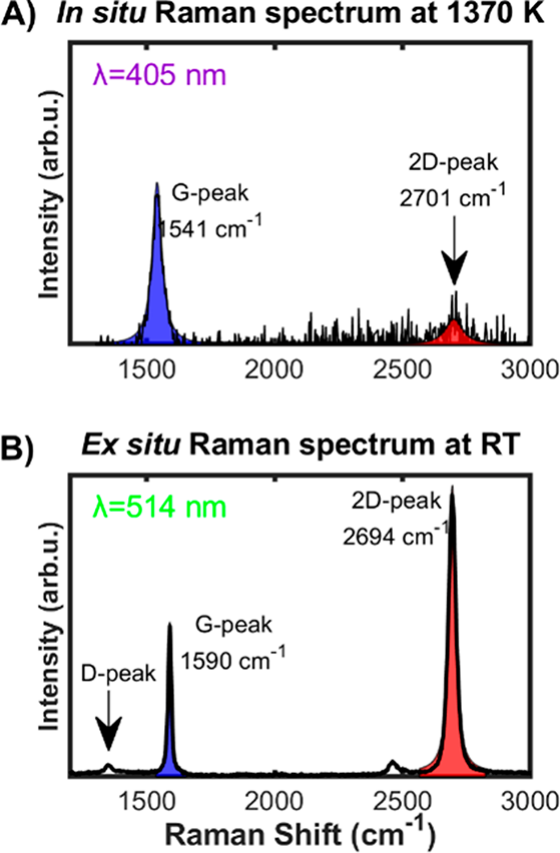
*In situ* and *ex situ* Raman spectra of graphene. (A) *In situ* Raman spectrum of SLG growing on liquid copper at 1370 K with 405 nm laser line in order to minimize the background of blackbody radiation. (B) *Ex situ* Raman spectrum with 514 nm laser line on solidified copper after cooling to room temperature. See Methods section for Raman specifications.

**Figure 4:**
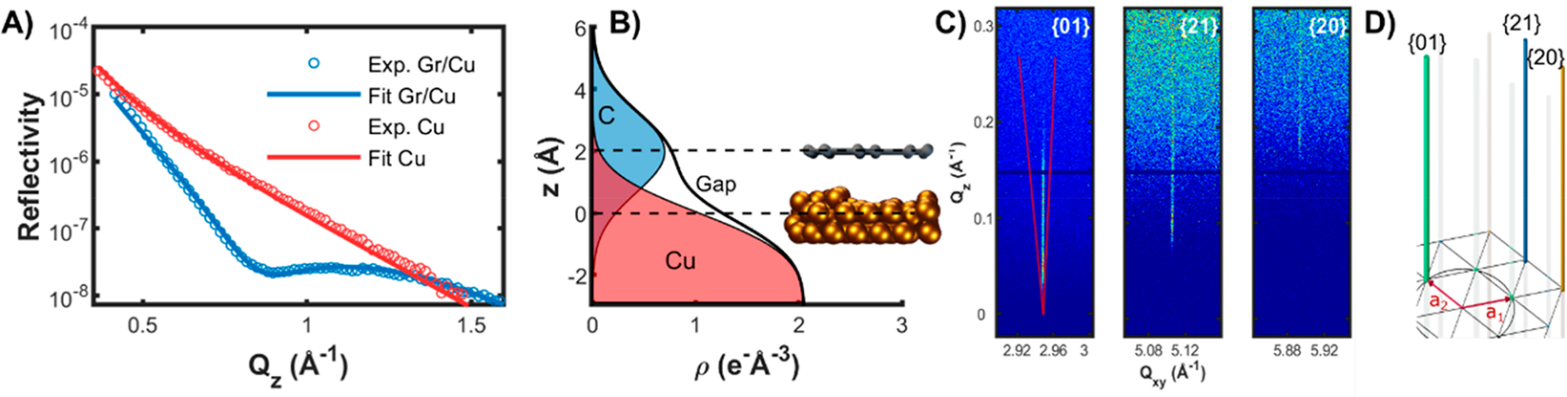
X-ray reflectivity and crystal truncation rods. (A) *In situ* XRR data for liquid copper with and without graphene overlayer. (B) Reconstructed electron density profile of SLG. (C) Position and fwhm’s of the Bragg rods as a function of *Q*_*z*_. The *Q*_*xy*_ distance between inclined lines on the {01} Bragg rod image indicates the fwhm of the corresponding Bragg rod of the exfoliated graphene measured with electron microscopy in ref (55). (D) Reciprocal-space lattice of graphene with {01}, {20}, and {21} rods.

To assess the chemical and atomic-scale structural properties of the growing graphene, we performed Raman spectroscopy (Figure 3A, B), XRR (Figure 4A, B), and GIXD (Figure 4C, D) measurements. *In situ* Raman spectra acquired during graphene growth confirmed that the grown film is indeed SLG (see Figure 3A and Supplementary Note 7). At elevated temperatures, a 405 nm laser line was implemented to acquire Raman spectra in order to minimize the blackbody radiation background, however, at the expense of 2D peak intensity. As mentioned earlier, *in situ* Raman has successfully been employed for obtaining Raman spectra *in situ* during Gr growth on molten Cu at elevated temperatures. In fact, such measurements are mandatory to assess the nature (SLG) and quality of the grown Gr. As expected, both 2D and G peak positions (∼2703 and 1542 cm^–1^, respectively) are significantly blue-shifted due to the anharmonic terms in the lattice potential energy, which are determined by the anharmonic potential constants, the phonon occupation number, and the thermal expansion of the crystal.^49^ The Lorentzian peak shape and the relative positions of the 2D and G peaks are characteristic for SLG.^50^ The presence of SLG is further supported by *ex situ* Raman spectroscopy (Figure 3B) with a ratio *I*_2D_/*I*_G_ ∼ 2, an average fwhm of ∼34 cm^–1^, and an extremely small *I*_D_/*I*_G_ ratio of ∼0.05, demonstrating low defect density. Detailed Raman mappings of individual graphene domains are presented in Supplementary Figure 6, which verify its hexagonal shape, and uniformity, and the residual stresses distribution over the flake. Differences in the *I*_2D_/*I*_G_ ratio between the *ex situ* and the *in situ* Raman spectra are mainly attributed to the background of the Raman spectra in the *in situ* measurements. At high temperatures, the substrate emits a significant amount of blackbody radiation, the intensity of which is proportional to the wavelength. For the case at hand, since the 2D peak lies at a higher wavelength compared to the G peak (455 nm *vs* 433 nm, respectively), the intense background engulfs the 2D peak, and therefore its full deconvolution is problematic.

*In situ* XRR measurements (Figure 4A) provide the out-of-plane electron density profile (Figure 4B), from which we confirm the formation of SLG, and we deduce low values of 1.2 Å for both Cu and graphene roughness, as well as 2 ± 0.1 Å for the Cu–C average separation distance when using the same definition of the distance as given above for the MD simulations. The latter simulations model a perfect graphene layer without defects and a pure liquid copper surface and give rise to a somewhat larger separation value of 2.89 Å.^51^ An explanation for the discrepancy between theory and experiment could be inaccuracies in the Cu–C interaction curve predicted by the employed force field and the presence of defects in the experiment. During CVD growth, graphene undergoes continuous defect formation (*e.g*., H_2_ attack) and self-healing. This could increase the 2DM/LMCat interaction energy and lower the average separation distance.^52^ In addition, the presence of trace impurities on the LMCat surface during CVD could also contribute to the discrepancy between the experimental and theoretical separation values. However, this factor seems less probable due to the high purity of our system (see Supplementary Note 8). *In situ* GIXD (Figure 4C,D) confirms the graphene’s superior crystallinity. As expected for SLG, the diffraction rods extend far perpendicular to the surface and have very small, resolution-limited widths, corresponding to crystallite sizes larger than 10 μm. For the large flakes, the Bragg rods are only visible in well-defined azimuthal directions, and for a given reflection type (*i.e*., given Miller indexes or Bragg angle), only one rod is recorded within 60° of azimuthal rotation, which confirms single crystallinity of the flakes. The precise position of several hundredths of Bragg rods provides the lattice constant (2.4601 ± 0.0005 Å at 1370 K). This value is slightly lower than the 2.4618 Å reported for graphite at the same temperature.^53,54^ At variance with the rods measured on suspended graphene sheets,^55^ the rods measured here do not show any increasing broadening in perpendicular *q*_*z*_ direction, proving the damping of microscopic corrugations of SLG on liquid copper.

### Real-Time Growth Tailoring

**Figure 5:**
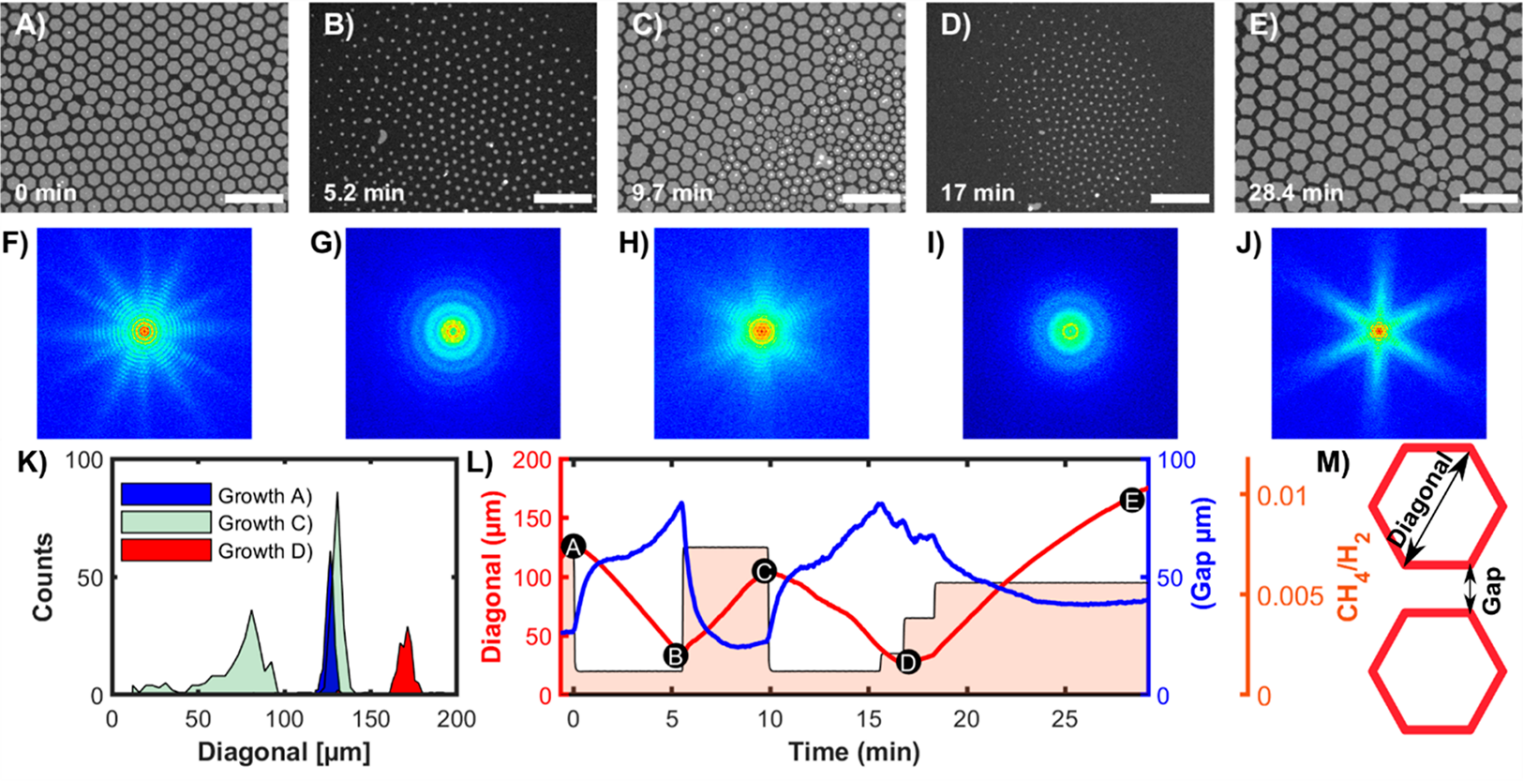
Real-time tailoring of graphene growth on liquid copper (see also Movie 2). (A)–(E) Optical microscopy images of graphene growth, etching, regrowth, second etching, and second regrowth on liquid copper. The Ar and H_2_ flow, pressure, and temperature correspond to 200 and 20 sccm, 0.2 bar, and 1370 K, respectively. The methane flow is varied. The relative time Δ*t* = 0 at the start of the first etching cycle is indicated. The length of the scale bars corresponds to 500 μm. (F)–(J) 2D FFTs of the images A–E, respectively.( K) Flake-size distributions in conditions A, C, and D. (L) Time evolution of flake diagonal (red), the average distance between flakes (blue), and methane flow (pink area). Marked are the times when frames A–E were recorded. (M) Schematic of definitions of the distance between flakes and the flake size (the diagonal).

*In situ* monitoring provides a possibility to vary the growth conditions and to observe the effects of these modifications in real time. This saves significant time and costs to find the right parameters to grow high-quality 2DMs and improves the reproducibility of the produced 2DM specifications. On the other hand, *ex situ* observations typically need multiple time-consuming trials to optimize the process while remaining inevitably vulnerable to the stochastic nature of growth mechanisms. An example of such real-time tailoring of the growth is presented in Figure 5A–E and Movie 2, in which the process is manipulated by alternating growth and etching stages in order to improve the flake ordering and size monodispersity. Initially, as shown in Figure 1 and Movie 1, the pulsed injection of CH_4_ resulted in many hexagonally ordered flakes (Figure 5A and before the first minute of Movie 2). However, the order is only short-range with two misaligned hexagonal networks. The azimuthal flake alignment is better revealed in the 2D Fast Fourier Transform (FFT, Figure 5F) that displays two star-like patterns with 6-fold symmetry, rotated with respect to each other. To improve the order, the formed flakes were partially etched (start at the first minute of Movie 2) by decreasing the CH_4_ pressure. This caused shrinkage of all flakes down to much smaller sizes (Figure 5B,G and ∼6 min 30 s of Movie 2), before resuming the growth at time 6 min 42 s, which leads again to large organized flakes (Figure 5C,K, until 10 min 50 s of Movie 2). However, because of a too fast increase of *p*(CH_4_), the regrowth of previously nucleated graphene flakes was accompanied by a second nucleation wave, resulting in a bimodal flake size distribution and only partial hexagonal order (Figure 5H). Contrary to the initially nucleated flakes, several multilayer seeds, seen as bright points in Figure 5C (10 min 40 s of Movie 2), decorate the newly nucleated flakes. A second etching period (by decreasing *p*(CH_4_)) starting at time 10 min 55 s resulted again in small disordered flakes (Figure 5D,I, 17 min of Movie 2). The methane pressure was then gradually increased, resulting (Figure 5E and end of Movie 2 after 29 min and 30 s of growth) in the formation of a near-perfect hexagonal network of almost monodisperse flakes (red histogram in Figure 5K). The sharp streaks of the 6-fold star-like FFT (Figure 5J) confirm the high degree of order and symmetry. Figure 5L shows the evolution of the average flake size and gap. This sequence illustrates how the growth can be manipulated in real time to arrive at an optimum configuration while starting from random and unsatisfactory ones. However, even here, some defects between the flakes remain upon coalescence. We found no strategy to avoid domain boundary defects altogether, despite the systematic variation of growth parameters while monitoring *in situ*. Therefore, we speculate that these domain boundaries are an intrinsic thermodynamic feature, likely induced by misaligning forces due to higher-order electrostatic interactions between the somewhat more charged corners of the flakes.

**Figure 6:**
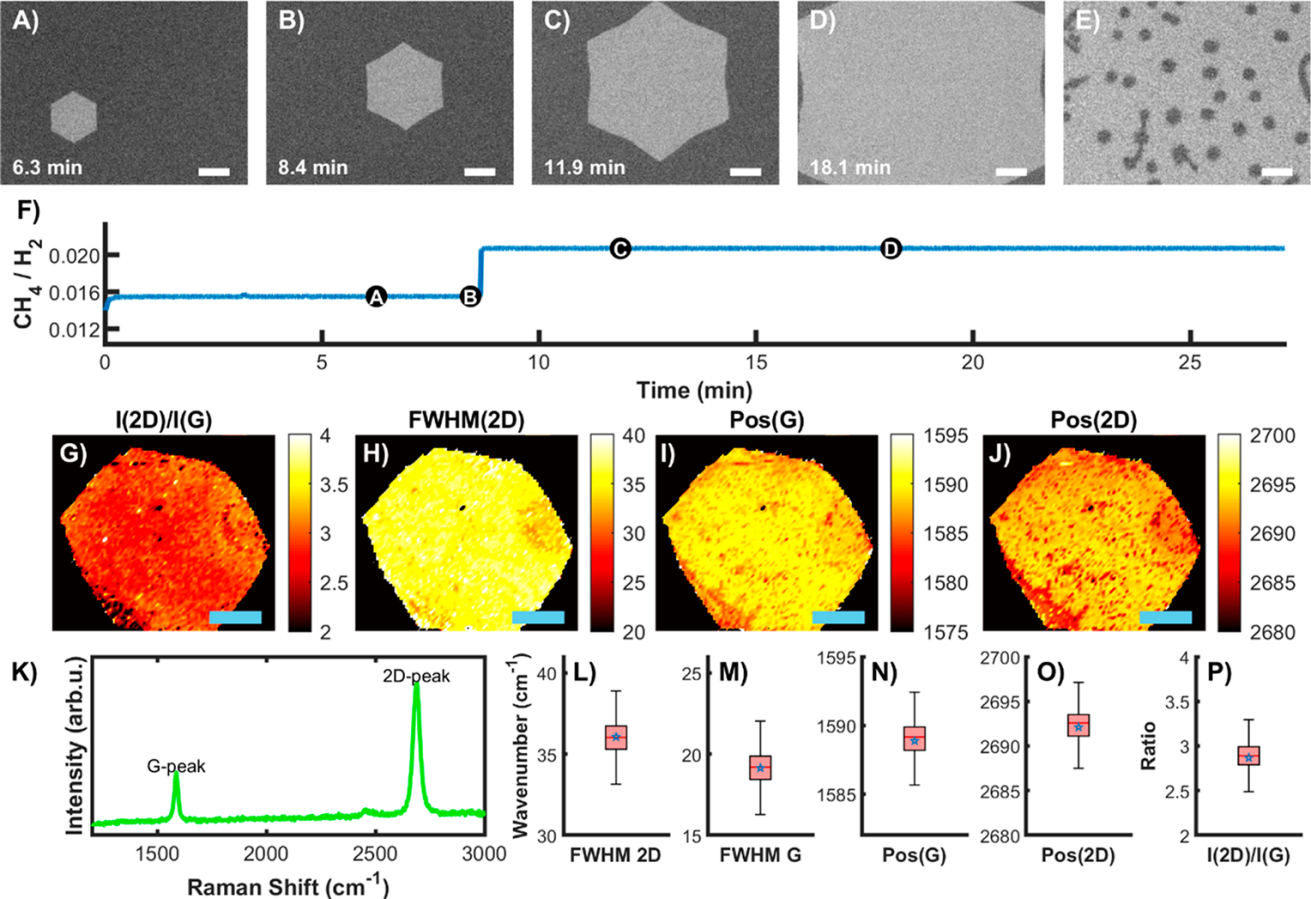
Single-crystalline graphene growth on liquid copper (see also Movie 3). Time lapse of graphene growth (A)–(D) and subsequent etching (E) recorded by *in situ* optical microscopy. The length of the scale bars corresponds to 100 μm. (F) CH_4_/H_2_ pressure ratio for the growth sequence in (A)–(D). (G)–(J) Raman map of *I*_2D_/*I*_G_, fwhm(2D), Pos(G), and *I*_D_/*I*_G_ recorded *ex situ* after cooling to room temperature. The scale bar corresponds to 200 μm. (K) Raman spectrum with characteristic G and 2D peaks. (L)–(P) box plots for FWHM(2D), FWHM(G), Pos(G), Pos(2D), and *I*_2D_/*I*_G_.

To circumvent this effect, we switched to radically different growth protocols that aim at nucleating and growing only one seed/flake on the entire surface. As shown in Figure 6A–D and Movie 3, a decreased nucleation rate leading to the eventual growth of one or very few large flakes can be achieved by tuning the influx of methane (Figure 6F). Initially, setting a small ratio of methane to hydrogen pressure of *p*(CH_4_)/*p*(H_2_) ∼ 0.015 resulted in the lack of any visible nucleation events. Only after a few minutes, the nucleation (not shown) of a single flake having the shape of a hexagon with rounded corners was observed. After 8 min, the ratio was increased to *p*(CH_4_)/*p*(H_2_) ∼ 0.02 in order to increase the growth speed. The nucleated flake gradually transformed into perfectly hexagonal after about 12 min of growth (start of Movie 3, Figure 6A) and later to a concave equilateral dodecagon (Figure 6C). After 24 min of growth, the size exceeds the field of view of millimeter size, corresponding to an average growth speed of ∼1 μm/s. The final macroscopic size is only limited by the area of the liquid Cu pool. In contrast to the previous multiflake growth case, when we now tune the *p*(CH_4_)/*p*(H_2_) ratio to etching conditions, we observe only slow etching at random points on the flake (Figure 6E). This indicates that the internal lattice imperfections are effectively limited to point defects (*e.g*., vacancies or Stone–Wales defects), while more extended defects like domain boundaries are absent. Extensive Raman mapping (Figure 6G,H) shows the high homogeneity of the final giant flake, and all characteristic average values (*e.g*., *I*_2D_/*I*_G_ of ∼3, fwhm of 2D peak of ∼35 cm^–1^) confirm its single-layer character. The lack of observation of the D peak confirms that the atomic defect density is below the detection level. Furthermore, at the resolution of the mapping step (10 μm), the flake appears to be continuous without domain boundaries. The average residual biaxial strain^56^ is minimal (∼0.1%) thanks to graphene’s weak bonding to liquid Cu (Supplementary Figure 9). Finally, the sheet resistance and the electrical conductivity were measured using the Van der Pauw method^1^ (see Supplementary Note 9) and found to be around 280 Ω/sq and 1.1 × 10^7^ S/m, respectively. These values are much higher than those obtained from the state-of-the-art graphene growth *via* conventional CVD on solid metal catalysts^57^ and are similar to those obtained from exfoliated flakes.^58−60^ Therefore, we conclude that the grown graphene film is of comparable quality to those obtained by tape exfoliation.^61^

## Conclusions

In summary, we demonstrated *in situ* multiscale monitoring of graphene growth on liquid copper *via* optical microscopy, Raman spectroscopy, GIXD, and XRR, and tailoring of the graphene growth thanks to direct feedback on growth parameters according to the observed changes in morphology, structure, or defects. The experimental observations are supported by multiscale modeling of short- and long-range interactions between graphene flakes, explaining their movement and assembly into a 2D hexagonal network of single-crystal graphene on liquid copper. Three examples of such tailored growths are given. In the first one, fast growth from multiple nucleation seeds results in single-layer but polycrystalline graphene sheets with domain boundaries. In a second example, we showed that adjusting the pressures of the different gases (here hydrogen and methane) in real time allows for selecting the flake shape, improving their size monodispersity, and producing highly ordered flake assemblies during just one growth trial. In the last example, we show that starting from a single nucleation seed instead results in a macroscopic, single-crystalline, and single-layer graphene sheet of superb quality.

The presented examples demonstrate the power of real-time control for the growth of 2DMs, which can also be implemented for the scientific study or industrial production of other nanomaterial classes, *e.g*., h-BN,^62^ GaN,^29^ or ultrathin oxide layers.^30^ With a growth speed of about ∼1 μm/s, a sheet resistance of 280 Ω/sq, and superior crystalline quality, this process is practically viable and allows for single-crystal production suitable for different electronic applications. Since the viscous forces that hold the grown material on the liquid metal are extremely weak compared to frictional forces on a solid, this process may also be suitable for an ambitious goal: continuous production. In this process, one can imagine the forming graphene sheet gradually pulled away from the liquid copper without cooling to room temperature, thereby preventing the wrinkling and folding due to differences in graphene and substrate material thermal expansion.

## Methods

### Experimental Setup

All experiments were performed using a setup suitable for the growth of graphene on liquid copper using chemical vapor deposition. The growth can be monitored *in situ* using X-ray-based techniques (Grazing-incidence X-ray diffraction (GIXD) and X-ray reflectivity (XRR)), Raman spectroscopy, and optical microscopy.^63^ The reactor has a cylindrical shape with a wall consisting of X-ray-transparent beryllium. A quartz window is placed on top of the reactor providing optical access for the Raman spectroscopy and optical microscopy measurements (see below). A custom-made heater assembly, capable of providing temperatures up to 1600 K, is placed at the center of the reactor. The sample holder consists of a tungsten disk in direct contact with the heater. Copper is added to the sample holder in the form of ultrapure foils. Copper is molten by heating the foils to 1370 K. A custom-built gas handling system is used to mix reactant gases (CH_4_ and H_2_) with Ar background gas with controllable mass flow ratios, and to deliver the gas mixture to the reactor.

### Graphene Growth on Liquid Copper

Graphene is grown on liquid copper (*T* = 1370 K) *via* chemical vapor deposition. Using the gas handling system, a 2% mixture of CH_4_/Ar and H_2_ is added to the reactor at a total pressure of 200 mbar. The flow rates are 200 sccm for Ar, 5–20 sccm for H_2_, and 0.1–20 sccm for CH_4_, respectively. The CH_4_ component dissociates catalytically on liquid copper, and the released carbon atoms form a graphene layer on the surface.

### Raman Spectroscopy

Raman spectroscopy has been successfully employed to characterize the growth of graphene on liquid copper. This technique is known to detect traces of precursor adsorbates, intermediate reaction species, and the grown graphene characteristics, such as the number of layers, the stacking type, the defect density, and the presence of dopants and/or contaminants. A 30 mW violet solid-state laser with an excitation wavelength of 405 nm is used for the Raman measurements in order to reduce the effects of blackbody radiation due to the high temperatures. The probe is equipped with microscopic long-working-distance objectives and is mounted on a motorized XYZ positioning system to adjust the positioning of the Raman objective lens in the optical port. To filter out the excitation laser light, a 405 nm edge filter was used. For *in situ* measurements, the scattered light was collected through the Raman microscope using a superlong-working-distance objective (50×, numerical aperture 0.35).

### X-ray-Based Measurements

The grazing-incidence X-ray diffraction (GIXD) experiments were performed at ID10 (ESRF, Grenoble, France). The monochromatic X-ray beam (*E* = 22 keV, λ = 0.056 nm, and Δ*E/E* = 1.4 × 10^–4^) was deflected downward to the liquid copper surface *via* a double crystal deflector (DCD) using Ge(111) and Ge(220) Bragg reflections. The grazing-incidence angle was set at 2.1 mrad, which corresponds to 80% of the critical angle of total reflection on the Cu surface at this energy. The beam size was 13 × 250 μm^2^ (*V* × *H*). Two-dimensional diffraction patterns, *e.g*., GIXD signals, were recorded using a Maxipix detector with a 1 mm thick CdTe sensor and 516 × 516 pixels of 55 μm in size. The detector was placed 922 mm downstream of the sample, and a vacuum flight tube was used to reduce air absorption and scattering. The detector size allowed measuring in one shot a reciprocal-space area of 0.03 × 0.03 nm^2^. Each family of graphene Bragg rods was measured by placing the detector at the expected in-plane scattering vector (*Q*_*xy*_). Several strategies to detect the graphene Bragg rods were applied: (i) time scans with a step of 0.5 s to probe the azimuthal-rotation dynamics of the graphene crystals and (ii) azimuthal rotation of the detector in the range −30° to +35° with steps of 0.05° to probe the orientation of the graphene crystals. Several hundreds of graphene Bragg rods on liquid copper were measured *in situ* at different growth conditions.

The X-ray reflectivity (XRR) measurements were performed at the P08 beamline of PETRA III (DESY, Hamburg, Germany). This beamline is equipped with a double crystal deflector (DCD) suitable for studies on liquid surfaces. The DCD deflects the X-ray beam downward to the horizontal surface using Bragg reflections from the Si(111) and Si(220) atomic planes. The beam size of X-rays of 18 keV photon energy (wavelength 0.0688 nm) was 40 μm × 200 μm (*V* × *H*). The reflected beam was measured with a 2D detector (Lambda, GaAs sensor) located at 1085 mm from the sample. Prior to the XRR modeling, the raw experimental data were background-subtracted. The obtained curves were fitted with the REFL1D program. Our model includes the liquid copper substrate, the graphene layer, and a gap in between. Variable parameters of the fit were the gap thickness and the roughness of the copper and graphene interfaces. All other parameters (electron density of copper and graphene, and graphene layer thickness = 1.42 Å) were fixed.

### Optical Microscopy

The sample surface was observed using a custom-built digital optical microscope. It consists of long-working-distance, infinity-corrected objectives with magnification options of 5×, 10×, 20×, and 50×, a tube lens with magnification 0.5×, and a CMOS-based digital camera. The system is capable of recording 30 frames per second with a maximum resolution of 4096 × 3000 pixels. The movies of the liquid copper sample and the growth of graphene on top of it were recorded in the radiation mode; *i.e*., there was no light source illuminating the sample, but only light radiated from the sample at high temperature was recorded. The observed contrast is caused by an emissivity difference between graphene and liquid copper, and the absorption of light passing through the graphene layer. The movies were recorded using Micro-Manager and MATLAB software. The frame rate was set between 0.2 and 5 frames per second. After the acquisition, the movies were digitally corrected using flat-field correction. The exposure time of the camera was usually set at 2–5 ms. Using the lowest-magnification lens, the observed area was 4980 × 3645 μm^2^, as deduced from calibration using the Multi-Frequency Grid Distortion Target from Thorlabs, which corresponds to 4096 × 3000 camera pixels. The calculated spatial resolution was ∼2 μm. The images were processed using MATLAB software. To reduce the size of the movies, frames were 2 × 2 or 4 × 4 binned, in order not to exceed a resolution of 1024 × 750 pixels. The global-intensity threshold of each movie was adjusted to obtain the best contrast between liquid copper and graphene flakes. For the final video production, the frames were exported to raw AVI format using MATLAB, followed by conversion to mp4 files and compression by the H.264 code using FFmpeg software. The statistical information about the graphene flakes is obtained after individual processing of each movie frame (see above). The images are converted to binary format, where value zero corresponds to the intensity of liquid copper, and value one to the intensity of graphene. The distance between the flakes is the distance between the centers-of-mass of the neighboring graphene flakes, and the gap value (the distance between neighboring flakes) is extracted from a linear profile connecting those centers-of-mass.

## Supporting Information Available

Movie illustrating a typical “pulsed” growth leading to an assembly of hexagonal flakes with a hexagonal order, as presented in Figure 1A–E (MP4)
Movie illustrating how the growth can be manipulated by successive sequences of growth and etching, as presented in Figure 2A–E (MP4)
Movie illustrating to the growth of a single large flake as discussed in Figure 3A–E (MP4)
Notes (1) concept of continuous 2DM production on LMCats, (2) growth conditions, (3) radiation-mode optical microscopy, (4) mechanism of 3D graphitic structures growth, (5) revealing graphene domain boundaries and defects by hydrogen etching, (6) theoretical framework, (7) assignment of Raman peaks of graphene, (8) purity of the produced graphene, (9) Van-der-Pauw method; Figures (1) profile of the methane gas pulse injected into the main stream of gas, (2) optical microscopy image of a multilayer stack of graphene, (3) example of a “pulsed” growth mode where multiple flakes are grown with multilayer carbon stacks, (4) evolution of the average gap between growing hexagonal flakes as a function of their size, (5) snapshot recorded during etching of a graphene layer after coalescence of flakes, (6) Raman spectroscopy contours of single flake, (7) AES spectrum of a single-layer graphene, (8) XPS spectrum of a single-layer graphene sheet, (9) Raman spectroscopy contour of a giant flake’s residual strain, and (10) schematic of the van der Pauw configuration (PDF)
