# Correction to Real-Time Multiscale Monitoring and Tailoring of Graphene Growth on Liquid Copper

**DOI:** 10.1021/acsnano.1c05447

**Published:** 2021-07-09

**Authors:** Maciej Jankowski, Mehdi Saedi, Francesco La Porta, Anastasios C. Manikas, Christos Tsakonas, Juan S. Cingolani, Mie Andersen, Marc de Voogd, Gertjan J. C. van Baarle, Karsten Reuter, Costas Galiotis, Gilles Renaud, Oleg V. Konovalov, Irene M. N. Groot

It has come to our attention that we did not correctly acknowledge the synchrotron where some of the measurements were performed. To rectify this, the acknowledgments section of our paper has been updated.

