# Supplementary material for: Real-Time Multiscale Monitoring and Tailoring of Graphene Growth on Liquid Copper
Source: ACS Nano. 2021 Jun 1;15(6):9638–48. doi: 10.1021/acsnano.0c10377 (PMC8291761; doi:10.1021/acsnano.0c10377)
Supplement: Supplementary file 4 — nn0c10377_si_004.pdf [file nn0c10377_si_004.pdf]

Supplementary Materials for

Real-Time Multiscale Monitoring and Tailoring of Graphene Growth on Liquid Copper

Maciej Jankowski[1,3, a], Mehdi Saedi[2*, a], Francesco La Porta[3], Anastasios C. Manikas[4], Christos Tsakonas[4], Juan S. Cingolani[5], Mie Andersen[5], Marc de Voogd[6], Gertjan J. C. van Baarle[6], Karsten Reuter[5], Costas Galiotis[4], Gilles Renaud[1], Oleg V. Konovalov[3], Irene M. N. Groot[2*]

[1] *Univ. Grenoble Alpes, CEA, IRIG/MEM/NRS 38000 Grenoble, France*

[2] *Leiden Institute of Chemistry, Leiden University, P.O. Box 9502, 2300 RA Leiden, The Netherlands*

[3] *ESRF−The European Synchrotron, 71 Avenue des Martyrs, 38043 Grenoble, France*

[4] *FORTH/ICE-HT and Dept. of Chem. Eng., University of Patras, Patras 26504, Greece*

[5] *Chair for Theoretical Chemistry and Catalysis Research Center, Technische Universität München, Lichtenbergstr 4, 85747 Garching, Germany*

[6] *Leiden Probe Microscopy (LPM), Kenauweg 21, 2331 BA Leiden, The Netherlands*

[a] *These authors contributed equally to this work*

**Corresponding authors e-mail: i.m.n.groot@lic.leidenuniv.nl, a.saedi@lic.leidenuniv.nl*

This file includes:

Supplementary Notes 1 to 9

Supplementary Figures 1 to 10

Supplementary Movie Captions 1 to 3

Supplementary Notes

Supplementary Note 1: Concept of continuous 2DM production on LMCats

The hypothetical concept of continuous 2DM production on LMCats (*e.g.* graphene on molten copper) *via* direct separation relies on the idea of pulling or sliding graphene from the liquid substrate. [1]The experimentally measured ultimate tensile strength of CVD grown single-layer graphene (SLG) is 30–33 $[N\ \mathrm{m}^{-1}]$ at room temperature.[2] At the liquid copper temperature of 1370 K, this value is predicted to be reduced by ~30%.[3] To the best of our knowledge, the adhesion energy between SLG and liquid copper (caused by van der Waals interactions) has not been reported, but it is measured to be 0.3 $[J\ \mathrm{m}^{-2}]$ between graphite and liquid copper.[4] The adhesion energy of SLG on liquid copper is expected to be slightly less than the one of graphite, as the weak van der Waals contributions of subsurface layers in graphite are absent in the case of SLG. Hence, the average force per unit width needed to separate the SLG off the molten copper directly is predicted to be about or less than 0.3 $[N\ \mathrm{m}^{-1}]$, which is about two orders of magnitude less than its tensile strength. However, one should note that the strength of graphene is a sensitive function of its defects (*e.g.*, vacancies, domain boundaries, cracks).[2,5,6] Therefore, *in situ* monitoring of graphene's structural quality during its growth (from atomic to macroscopic scale) is of outmost importance for a successful direct separation process.

Supplementary Note 2: Growth conditions

We have grown graphene on a liquid copper surface using a continuous flow of an H_2/Ar/CH_4 gas mixture. The total pressure in the CVD reactor was usually set at 200 mbar, and the flow of gases at 200 sccm of Ar and 20 sccm of H_2. Under these conditions, the power of the sample heater was slowly raised until the melting of copper, observed by optical microscopy. The new copper samples were annealed for a few hours in a mixture of H_2 and Ar in order to remove impurities segregating at the surface, visible as bright features slowly moving to the edges of

the liquid copper pool. Subsequently, a gas mixture of 2% methane in argon was introduced to the reactor chamber at a flow of 7 sccm, which corresponds to a methane flow of 0.14 sccm. The initiation of the growth by a so-called “pulsed growth” was done by accumulating the methane-argon gas mixture for 40 s in the gas line and its sudden release to the mainstream of gas by opening the electromagnetic valve. The profile of the pulse is presented in Supplementary Fig. 1. The temporary increase of methane pressure was 22.5 times higher with respect to continuous flow. The gas composition in the main line connected to the reactor was measured using a residual gas analyzer (RGA) separated from the main line with an ultra-high vacuum (UHV) leak valve.

Supplementary Note 3: Radiation-mode optical microscopy

Radiation-mode optical microscopy is an experimental method allowing to monitor the growth of thin layers on surfaces at high temperatures. This method employs an optical microscope measuring the light radiated by the sample. The recorded microscopy images show areas with different intensities (see Supplementary Fig. 2). This contrast originates from sample regions exhibiting different emissivity[7]. The contrast between liquid copper and graphene layer(s) is caused by the interplay between the different emissivities of liquid copper and graphene (0.143 and 0.016, respectively) and the light absorption by the graphene layer (2.5% per layer).[8,9] One monolayer of graphene absorbs the light emitted by the liquid copper that supports it, but also emits light itself. The overall balance results in a theoretically 8% higher luminous emittance for SLG-covered rather than bare liquid copper above its melting temperature. Moreover, the emissivity of multilayer graphene scales linearly with the number of layers.[10]

Supplementary Note 4: The mechanism of 3D graphitic structures growth

At the initial stages of the growth, depending on the gas flow conditions, we observe multilayer carbon stacks in the center of the growing flakes (see Supplementary Fig. 2a and see Supplementary Fig. 3a). These stacks disappear in the final stages of the growth when graphene flakes merge and form a continuous layer. The observed phenomena can be explained by the mechanism proposed by Kalbac *et al.*,[11], which is confirmed by microscopy images presented in Supplementary Fig. 3. It has been proposed that the decomposition of the methane on the hot copper leads to the formation of graphitic carbon, in our case, multilayer carbon stacks. The growth rate of each layer in these stacks depends on the distance to the exposed LMCat surface, acting as a source of carbon atoms. The layers which are closest to the LMCat surface grow at the highest rate. As the bottom carbon layer grows, the distance between top layers and the source of carbon atoms increases, their growth slows down, to finally stop when the first layer is sufficiently large (see Supplementary Fig. 3b). The top layers are continuously attacked by the hydrogen present in the flown gas mixture during the whole growth. When the distance that needs to be travel by carbon atoms is too high, the layer's etching prevails over the growth, and finally, the multilayer-stacks gradually disappear (see Supplementary Fig. 3c and d). Also, this observation supports the idea of the initial growth of graphene from multilayer carbon seeds.

Supplementary Note 5: Revealing graphene domain boundaries and defects by hydrogen etching

Etching by the Hydrogen can reveal some defects of a fully-grown graphene layer during one single experiment. After the coalescence of growing graphene flakes, it is possible to determine if the grown layer is continuous, without extended grain boundaries and defects. The flow of the precursor gas, methane, is turned off, and the sample is exposed only to the mixture of Argon and Hydrogen, with the same flows used for the growth. The etching of graphene leads to the appearance of linear voids at the place of domain boundaries[12] and compact voids where

other defects are expected[13]. The effects of the initial etching are presented in Supplementary Fig. 5. From the presence and geometry of voids, it is possible to deduce information about the graphene layer's homogeneity.

Supplementary Note 6: Theoretical framework

Complementing the experiments, we provide a theoretical framework analyzing the interactions between growing graphene flakes. These interactions consist of attractive capillary interactions and repulsive electrostatic interactions. Interactions between particles on a fluid-fluid interface have previously been rationalized in terms of the deformation of the fluid surface around the particles as originating from the three-phase contact angle.[14–16] In this work, we specifically model such interactions within the general framework of capillary multipoles as formulated by Danov *et al.*,[17]where the formula for the force among two capillary charges is derived assuming a small meniscus slope between the particles. This leads to the following equation for the energy change, $\Delta W(L)$, when two particles are brought together to a distance L from infinity:

$\Delta W(L) = -2\pi\gamma Q_A Q_B K_0(L/l_c)$ (monopole–monopole), Eq. 1

where Q_A and Q_B are the capillary charges of particles *A* and *B*, K_0 is the modified Bessel function of second kind and order zero, l_C is the so-called capillary length and γ is the surface tension of the liquid. The capillary length is given by $l_C = \sqrt{\gamma/(\Delta\rho g)}$, where $\Delta\rho$ is the difference in density between the two fluid phases and g is the gravitational acceleration. l_C is calculated to be ~4 mm for Cu at 1370 K using this formula, the density from Assael *et al.*[18] and the surface tension from Matsumoto *et al.*,[19] The capillary charge can be related to the three-phase contact angle for spherical particles:[20]

$Q_X = \frac{r_X^3}{6\, l_c^2}(2 - 4D_X + 3\cos\alpha_X - \cos^3\alpha_X)$, Eq. 2

where r_X is the radius of particle X, α_X its contact angle, and D_X relates the densities of the different phases, here taken as the ratio of the particle and liquid densities. We here model the flakes as spherical particles with a radius of 70 μm, where the radius is chosen to match half of the diagonal of the flake sizes typically observed in the experiment. In turn, the contact angle is related to the liquid-particle interface energy $\gamma_{\mathrm{graphene-Cu}}$ by Young's equation:

$$\cos\alpha = \frac{(\gamma_{\mathrm{graphene-vapour}} - \gamma_{\mathrm{graphene-Cu}})}{\gamma_{\mathrm{Cu-vapour}}}, \qquad \text{Eq. 3}$$

where $\gamma_{\mathrm{graphene-vapor}}$ and $\gamma_{\mathrm{Cu-vapor}}$ are the surface energies of graphene and liquid Cu, respectively. The numerator in Eq. 3 was calculated by multiplying the graphene–Cu interface energy per atom obtained as discussed below by the graphene density of $0.382\ \mathrm{atoms}\ \text{Å}^{-2}$.

At the metallic surface, the doping of the graphene flake, as resulting from the equilibration of the Fermi levels,[21] is accompanied by the build-up of an image charge in the conductor. We here verify by finite-element calculations that the charge distribution inside the flake is essentially homogeneous, allowing us to approximate the repulsive electrostatic forces in our homogeneously-charged particle model as simple dipole interactions depending on the dipole moment formed by the doping charge and its image charge. A finite-element method following a similar approach to Georgantzinos *et al.*[22] was used to calculate the charge distribution inside a hexagonal graphene flake. The electrostatic potential is calculated at the center of each element by considering the rest as point charges and integrating the interaction within each element assuming a homogeneous distribution of the charge; that is, given the centers of the elements, $\mathbf{r}$, and their charges, $\mathbf{q}$, the electrostatic potential at the center of each element, $\mathbf{V}$, is given by:

$$\mathbf{V} = \mathbf{A}\mathbf{q} \qquad \text{Eq. 4}$$

$$\mathbf{A}_{ij} = \frac{1}{4\pi\epsilon_0}\frac{1}{|\mathbf{r}_i - \mathbf{r}_j|} \qquad \text{Eq. 5}$$

$$\mathbf{A}_{ii} = \frac{1}{4\pi\epsilon_0}\iint_S \frac{1}{s}\frac{1}{|\mathbf{r} - \mathbf{r}_i|}\mathrm{d}\mathbf{r}, \qquad \text{Eq. 6}$$

where the double integral runs over the interior region of the i-th element and s is its surface area. For hexagonal elements the self-interaction term is:

$$\mathbf{A}_{ii} = \frac{1}{4\pi\epsilon_0}\frac{2\log(3)}{R}, \qquad \text{Eq. 7}$$

where R is the radius of the circumscribed circle. A system of equations is then built to find the charge distribution that make all elements equipotential:

$$\mathbf{Aq} = \mathbf{I}V_0 \qquad \text{Eq. 8}$$

To account for the conductor surface which lies beneath the flake at a distance d, elements corresponding to mirror images of the charges are included in Eqs. 5 and 6 as an additional term where $d \cdot \hat{\mathbf{z}}$ is added inside the norm in the denominator. For the self-interaction case the corresponding integral was calculated numerically. We used hexagonal elements with an area of ~0.4 μm^2, resulting in 30,301 elements for a hexagonal flake, the side of which measures 70 μm. The number of elements were converged with respect to the electrostatic potential energy between two flakes at experimentally relevant distances. This particular size was originally chosen to match particular flake sizes observed in the experiment. However, calculations performed with other flake sizes show that the result, *i.e.* that the charge is homogeneously distributed, does not depend on the specific size of the flake.

Within the theoretical framework established above, the capillary-electrostatic model thus requires the estimation of only three microscopic parameters: the capillary interactions depend on the graphene–Cu interface energy and the electrostatic interactions depend on the dipole moment formed by the doping charge and its image charge, *i.e.* the two parameters required are the charge per carbon atom and the height of the flake above the liquid Cu surface. We obtained these parameters from molecular dynamics (MD) simulations of a liquid Cu surface covered by a graphene flake. We here consider a graphene flake where the edge atoms are not passivated by hydrogen, but by the Cu surface. This choice is motivated by our recent *ab initio* thermodynamics study[23] where we found that hydrogen- and metal-passivated edges have very

similar formation free energies under typical CVD growth conditions and that hydrogen-passivated flakes have very low adsorption energies as a result of weak van der Waals interactions with the Cu surface. Even if the formation of hydrogen-passivated flakes is thus possible, they would rapidly desorb at liquid Cu CVD temperatures, and we therefore do not consider such flakes in the present study.

The MD simulations were run using the LAMMPS[24] and ASE[25] codes using the COMB3[26,27] reactive interatomic potential. They consisted of a periodic boundary condition supercell geometry containing a graphene flake with zigzag edges adsorbed on one side of a Cu slab with an orthogonal (26 × 30) surface unit cell in the crystalline state and 9 (111) layers. The lateral dimensions of the supercell, generated with the optimized PBE[28] lattice constant, were scaled to their average, therefore producing a square simulation box of 67.0 Å by 67.0 Å. Simulations were run with different flake sizes containing up to 600 C atoms. The Cu slab was first melted and equilibrated using a Langevin thermostat set to 1380 K and with a 100 fs characteristic time for 2 ps. Then the system was pre-equilibrated with a Berendsen thermostat set to 1370 K with a timescale of 1000 fs for an extra 5 ps. After this, the flake was added above the slab and the same procedure was run again with the sole difference that the initial Langevin thermostat acted only on Cu atoms whereas the second thermostat acted on the entire system. The production run was carried out with a Nosé-Hoover thermostat set to 1370 K with a time constant of 50 fs. In all cases the time step was 1 fs. Properties were evaluated by first discarding a further 2 ps of the production run and then sampling every 0.1 ps for 100 ps.

For the distance between the flake and the Cu slab the full 100 ps were used, and the average was taken across different flake sizes. Given a trajectory, the inflection point of the mean density profile of Cu atoms under the flake (*i.e.* within the hexagon's inscribed circle) was used to define the liquid surface height, and the flake position was determined from the mean C position. The average height of the flake above the liquid surface was thereby found to be 2.86

± 0.10 Å. The charges were estimated based on the electronegativity equalization scheme of the COMB3 reactive interatomic potential. To estimate the error in the mean charge per atom, averages over blocks of 20 ps were used. Interface energies between flake and substrate were calculated by subtracting the energies of the isolated flake and isolated slab from the energy of the interacting system in snapshots from the MD simulations taken every 1 ps. Moreover, we assumed a simple scaling of the average charge per atom and interaction energy per atom with flake size by considering two contributions to the total charge or energy arising from the area of the flake, s, and its perimeter, l:

$$P = P_s s + P_l l, \qquad \text{Eq. 9}$$

where P is the property of interest. For a hexagonal flake the geometrical terms are related to the areal density of graphene by:

$$s = \frac{n}{\delta} \qquad \text{Eq. 10}$$

$$l = 6\left(\frac{2}{3\sqrt{3}\delta}\right)^{\frac{1}{2}} n^{\frac{1}{2}}, \qquad \text{Eq. 11}$$

where n is the number of atoms and δ is $0.382\,\frac{\text{atoms}}{\text{Å}^2}$. Finally replacing Eqs. 10 and 11 into Eq. 9 and dividing by the number of atoms, one can fit a linear model of the contribution per atom as a function of $1/\sqrt{n}$:

$$\frac{P}{n} = \frac{P_s}{\delta} + 6\left(\frac{2}{3\sqrt{3}\delta}\right)^{\frac{1}{2}} P_l \frac{1}{\sqrt{n}}. \qquad \text{Eq. 12}$$

The intercept of such a linear model corresponds to the asymptotic value for very large flakes, which is the one used in our model.

Following the above approach, we arrive at a charge transfer of 0.0445 ± 0.0001 electrons per C atom, which combined with the height estimated above yields a dipole moment of 1.22 ± 0.04 D per C atom. The total flake dipole moment is calculated by multiplying this number with the number of carbon atoms in a hexagonal flake, the side of which measures 70 µm. The asymptotic interfacial energy determined between graphene and liquid Cu is 95 ± 16 meV per

C atom, which is of comparable magnitude to the interfacial energy for graphene on solid Cu(111) reported from first-principles calculations.[23,29,30] Using these values in the capillary-electrostatic model derived above leads to an optimum distance of about 365 μm between two such particles. Superposition of the longer-ranged attractive capillary attractions in larger self-aligned ensembles of particles shrinks this optimum distance, for instance down to 102 μm for an assembly of 85 particles. We ascribe the remaining quantitative difference to the experimentally observed inter-flake distance of 40 μm to the simplicity of the model employed here. Apart from inadequacies of the molecular simulations (approximate interatomic potential, finite size of the simulation cell), we expect in particular higher-order capillary interactions arising for non-spherical particles,[14,31] as well as presently not considered electrocapillary interactions[16,32] to provide additional attraction that would further shrink the optimum inter-flake distance.

Supplementary Note 7: Assignment of Raman peaks of graphene

The Raman spectrum of graphene consists of three main peaks: the G peak (~1582 cm^{-1}), which is present in all sp^2 carbon materials and corresponds to the in-plane doubly degenerate E_g phonon at the center of the Brillouin zone, the D peak, which is due to the breathing modes of sp^2 rings, and is defect-induced. It is activated by a double resonant process of transverse-optical mode (TO) phonons around the K-point of the Brillouin zone, and the 2D peak (second order of the D peak) arising from a double resonant 2-phonon process and not requiring the presence of defects. The combination of the D, G, and 2D peak characteristics provides information about graphene such as: (1) defect density (I_D/I_G ratio), (2) number of layers through the full-width half maxima (FWHM) and shape of the 2D band, and the I_{2D}/I_G ratio), and (3) residual stress (*Pos*(G), *Pos*(2D) and *FWHM*(2D)).

Supplementary Note 8: Purity of the produced graphene

We present Auger electron spectroscopy (AES) and X-ray photoelectron spectroscopy (XPS) spectra recorded from the solid copper covered by graphene. The sample was cooled down to room temperature, removed from the CVD reactor, and quickly transferred to a UHV system equipped with a hemispherical electron analyzer, an X-ray gun, and an electron gun. Before measurements, the sample was degassed at 250 °C for a few hours in UHV. The characteristic peaks of copper are visible in the recorded AES spectrum, shown in Supplementary Fig. 7. The three KLL carbon peaks at ~270 eV confirm the presence of the graphene layer. The peak at 152 eV (S $L_3M_{23}M_{23}$) indicates the presence of residual traces of sulfur, which is an intrinsic impurity of copper or might be adsorbed on the sample surface during transport between the reactor and UHV system. The XPS spectrum (Supplementary Fig. 8) shows the presence of the carbon (C 1s peak) but not sulfur, which peak position (S 2p) corresponds to 164 eV. This peak absence indicates the presence of sulfur only in the surface region as AES has much higher surface sensitivity. For higher concentrations in bulk, we should be able to detect sulfur using XPS. In summary, the presented spectra show high chemical purity of the samples.

Supplementary Note 9: Van-der-Pauw method

Electrical properties of produced graphene samples were measured through the van der Pauw method which is a common way to measure resistivity. The main advantage of this method is the ability to measure the properties of a sample of any given shape if it is approximately two-dimensional. It uses a four-point probe positioned on the sample and provide the average

resistivity of the sample under the examined area. A scheme of the van der Pauw configuration is presented in Supplementary Fig. 10 with a probe spacing of around 2 mm.

Once all the voltage measurements are performed, two values of resistivity ρ_A and ρ_B are derived as follows:

$$\rho_A = \frac{\pi}{ln2} f_A t \frac{(V_1 - V_2 + V_3 - V_4)}{4I}$$

$$\rho_B = \frac{\pi}{ln2} f_B t \frac{(V_5 - V_6 + V_7 - V_8)}{4I}$$

where t is the thickness in cm, V1-V8 are the measured voltages, I is the applied current and f_A, f_B are geometrical factors based on sample symmetry. They are related to the two resistance ratios QA and QB as shown in the following equations:

$$Q_A = \frac{V_1 - V_2}{V_3 - V_4}$$

$$Q_B = \frac{V_5 - V_6}{V_7 - V_8}$$

where Q and f are corelated through the below equation.

$$\frac{Q - 1}{Q + 1} = \frac{f}{0.693} arc \cosh(\frac{e^{0.693}}{2})$$

Once ρ_A and ρ_B are calculated, the average resistivity was determined by:

$$\rho_{AVG} = \frac{\rho_A + \rho_B}{2}$$

Supplementary Figures

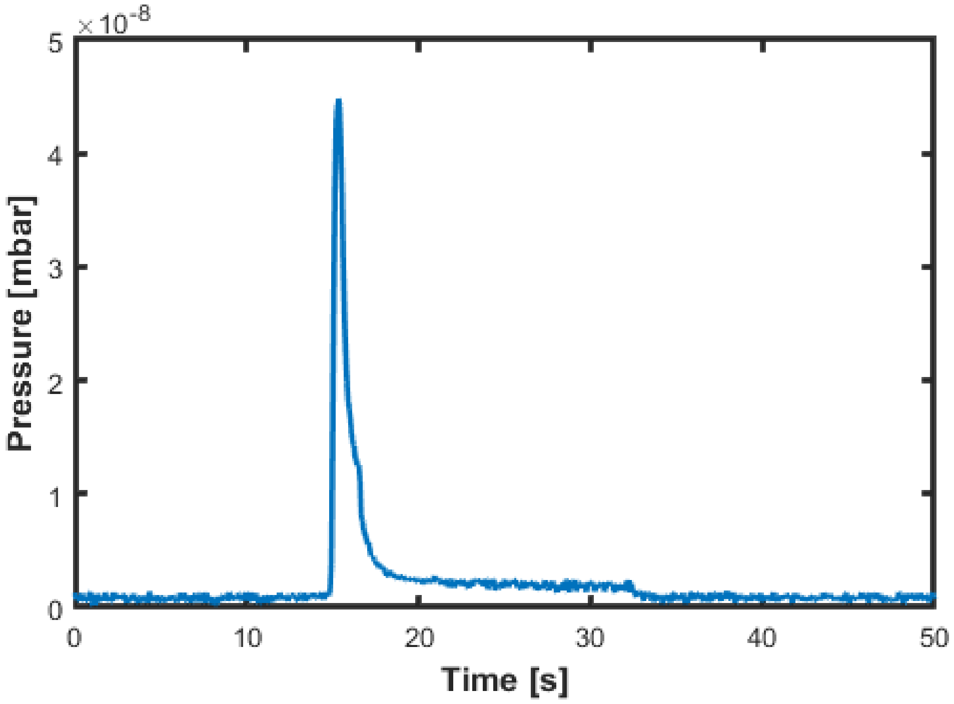

Supplementary Fig. 1:

The profile of the methane gas pulse injected into the main stream of gas.

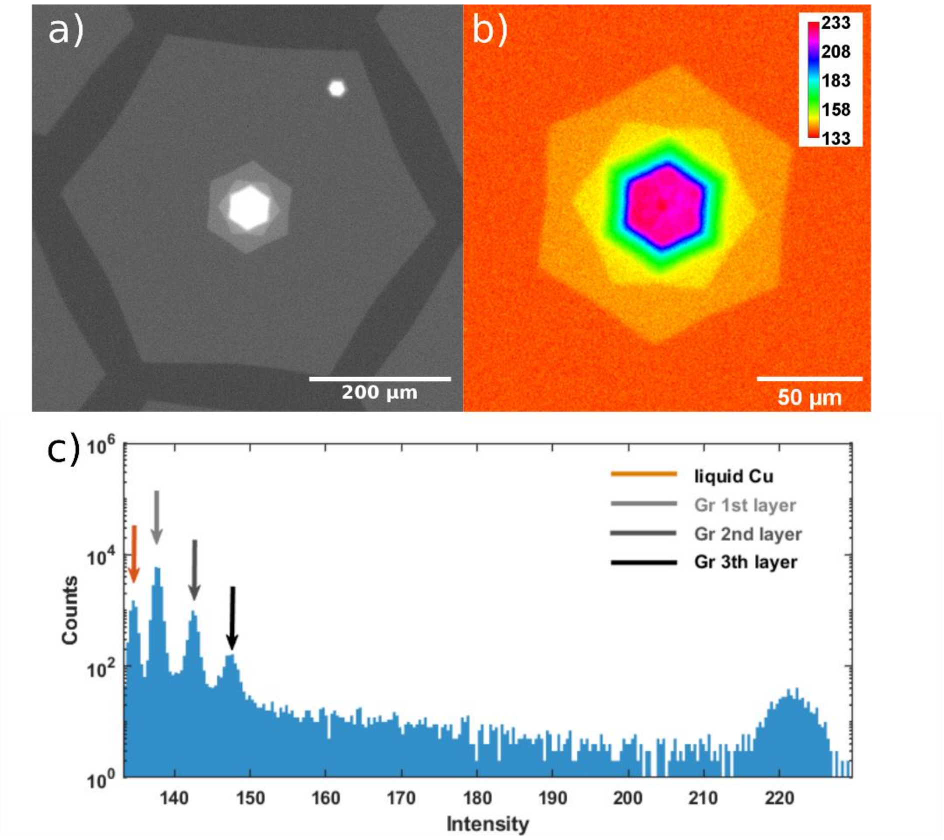

Supplementary Fig. 2:

a) Optical microscopy image of a multilayer stack of graphene at the center of a large graphene flake. b) The center of the stack is presented using a color scale where the color values correspond to the measured photon counts. c) The image-pixel-intensity histogram presented on a logarithmic scale. The arrows mark intensity levels of liquid copper and the first three layers of graphene.

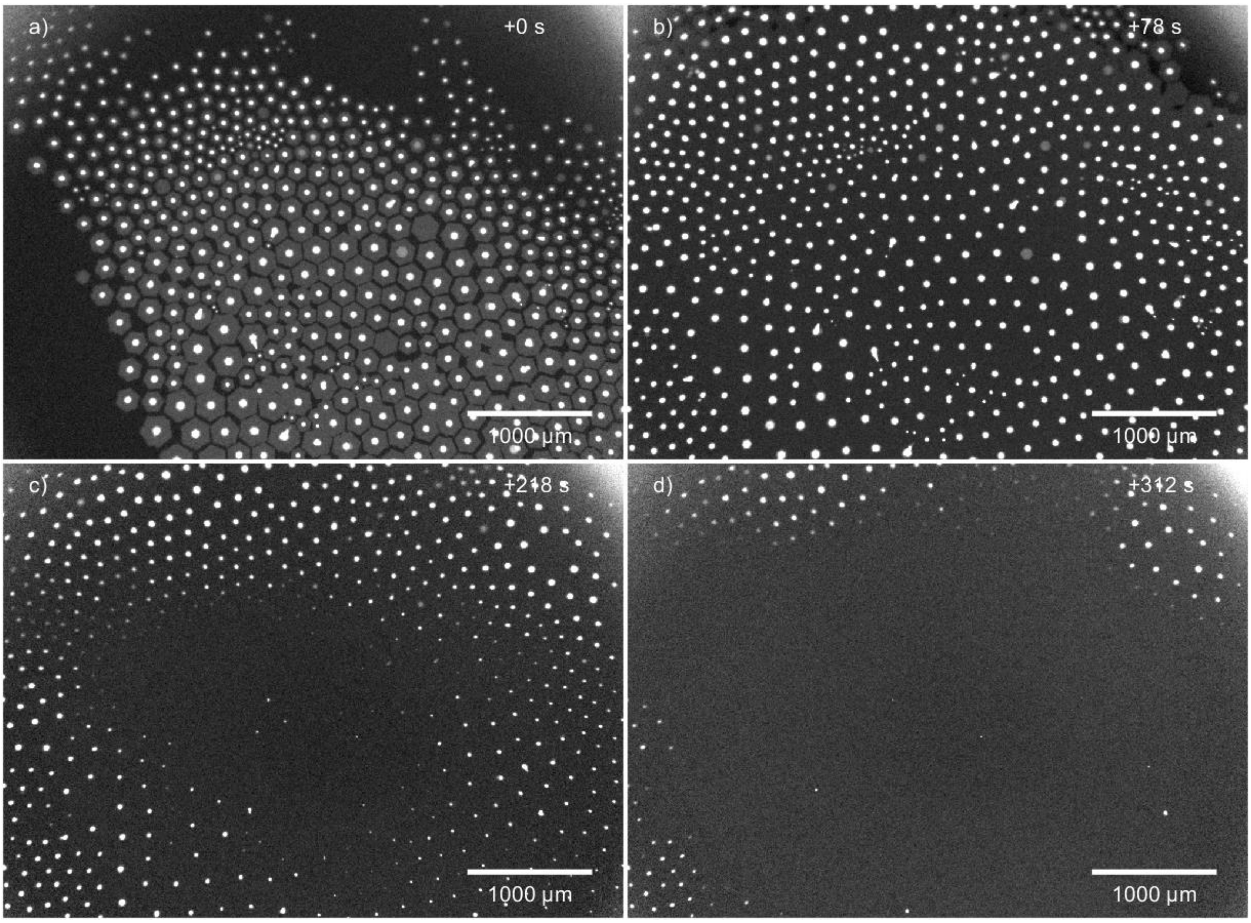

Supplementary Fig. 3:

A) Example of a ‘pulsed’ growth mode where multiple flakes are grown with white centers indicating multilayer carbon stacks. B) Almost entirely closed layer decorated with the carbon stacks. C) After merging flakes and layer closing, the gas flow conditions are not changed, and we observe the disappearance of the carbon stacks. D) Late stage of the growth where the multilayer carbon stacks are almost entirely etched while the gas flow is maintained at this same value as at the beginning of the growth.

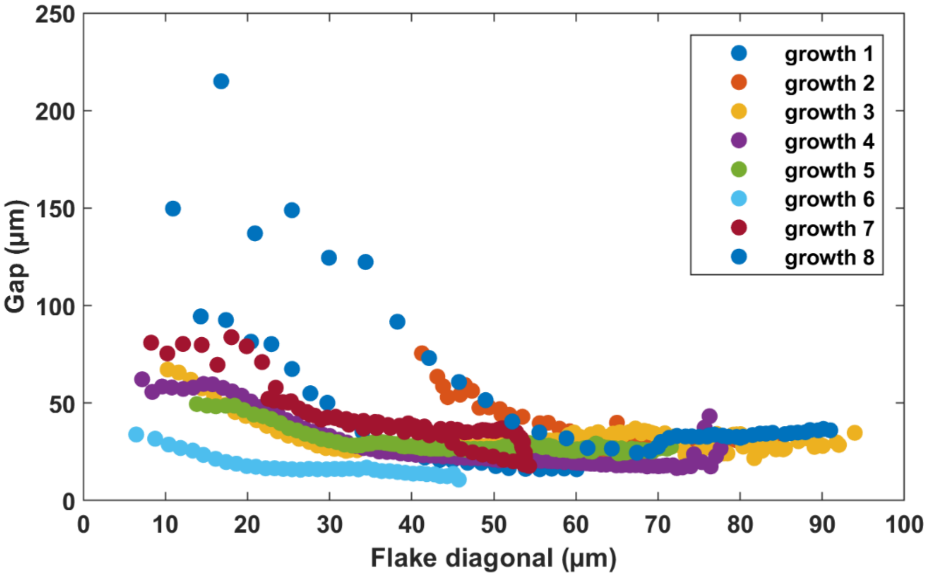

Supplementary Fig. 4:

Evolution of the average gap between growing hexagonal flakes as a function of their size (flake diagonal) for different pulsed growths. After an initial decrease depending on the growth, when the average flake area exceeds a size of typically 50–90 µm, the average gap value tends to a constant ~20 to 40 µm.

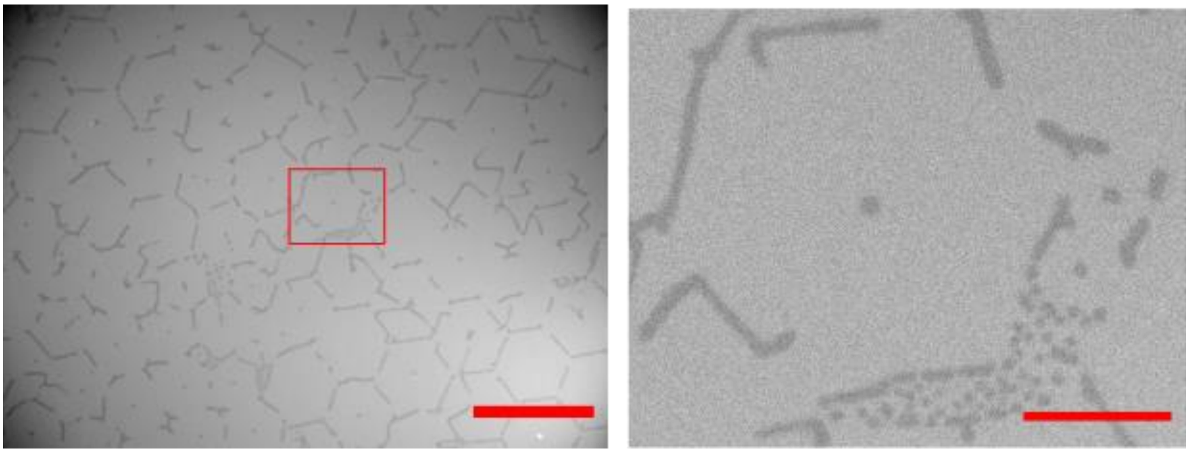

Supplementary Fig. 5:

Left: Snapshot recorded during etching of a graphene layer after coalescence of flakes. The length of the scale bar corresponds to 500 µm. Right: zoom-in on the area in a) marked with

the red rectangle. The 12 MP CMOS camera allows visualizing the surface with a maximum field of view of 5 mm and resolving small details with µm resolution, limited by the visible light's diffraction limit. The length of the scale bar corresponds to 100 µm.

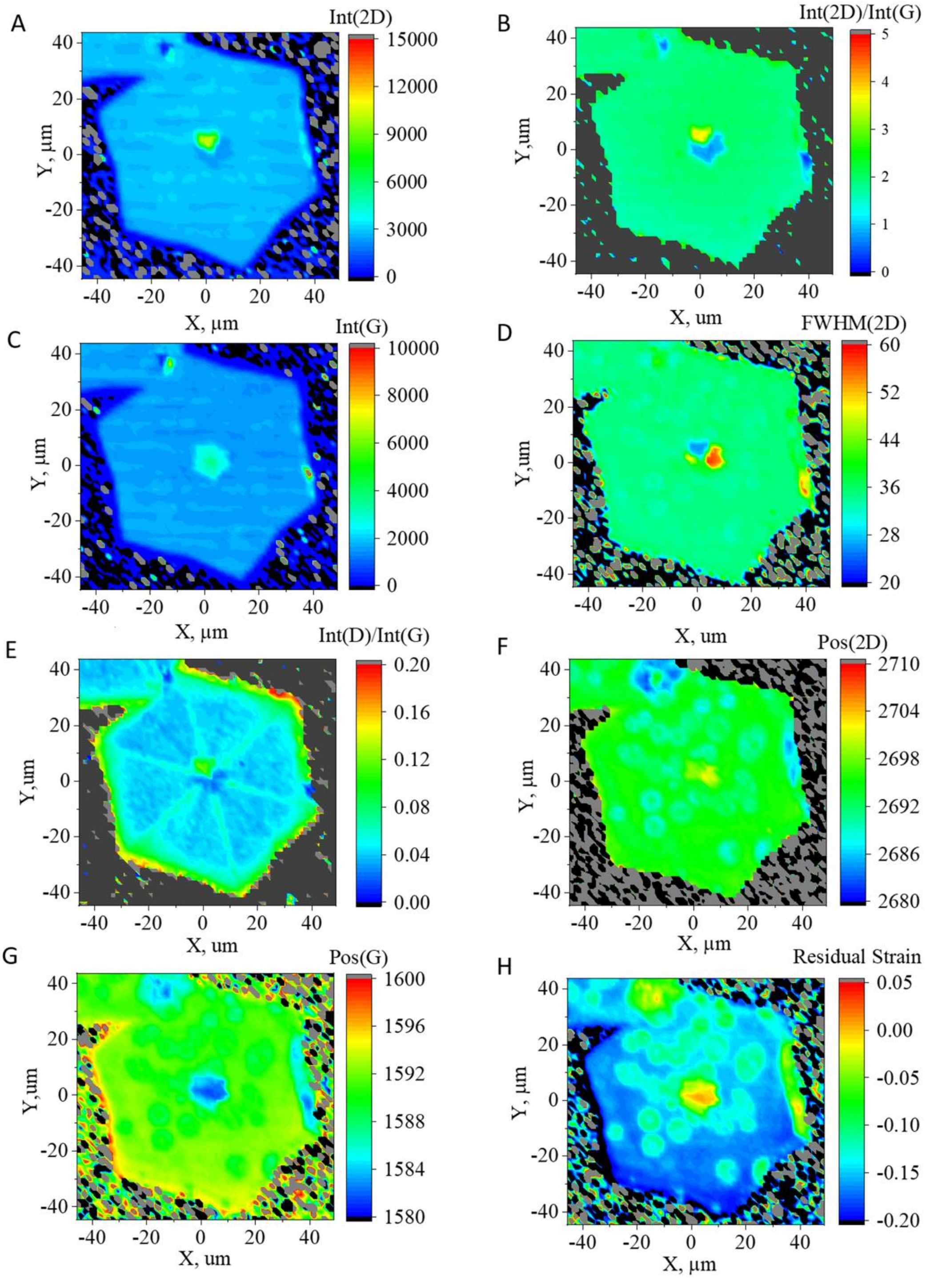

Supplementary Fig. 6: Raman spectroscopy contours of: A) Intensity of 2D peak; B) Ratio of of 2D to G peak intensities; C) Intensity of G peak; D) full width at half maximum of the 2D peak; E) Ratio of the D to G peak intensity; F) Position of 2D peak; G) Position of G peak; H) flake's residual strain.

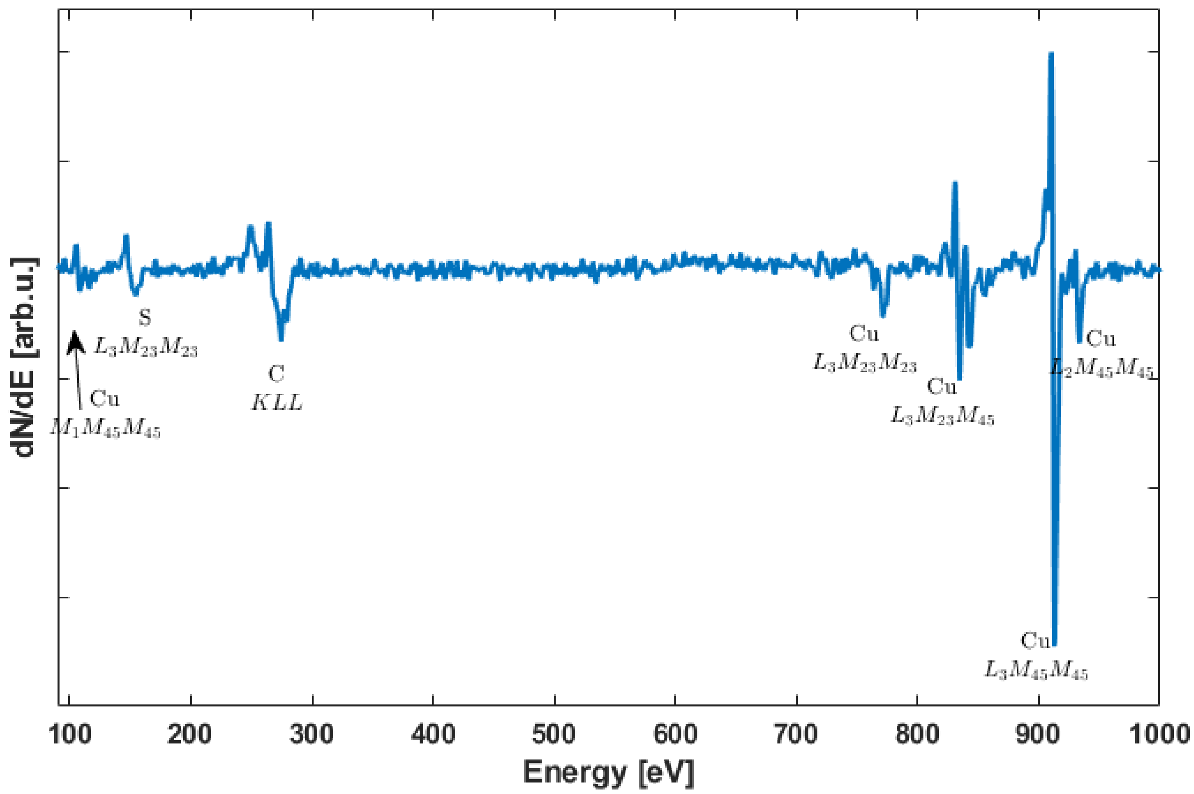

Supplementary Fig. 7:

AES spectrum of a single-layer graphene sheet grown on liquid copper after solidification and transfer to a UHV chamber. The energy of incident electrons is 4 keV.

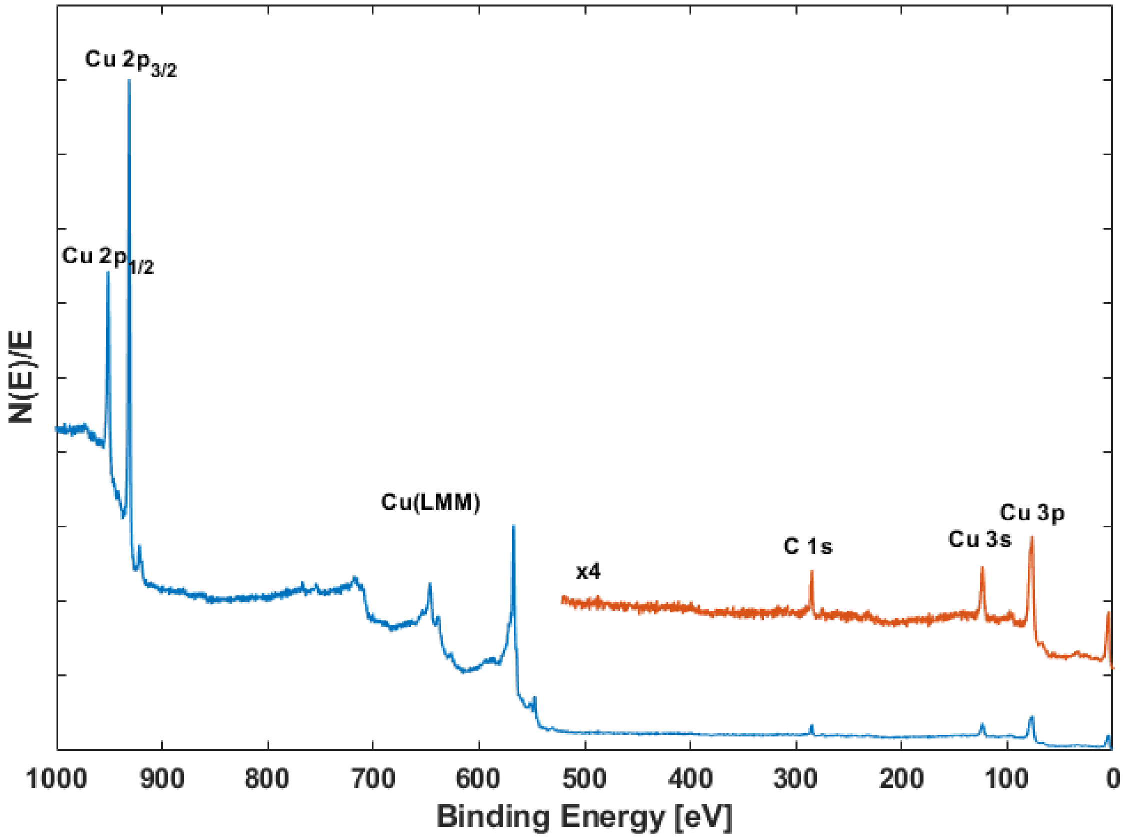

Supplementary Fig. 8:

XPS spectrum of a single-layer graphene sheet grown on liquid copper after solidification and transfer to a UHV chamber. The sample was measured using an Al Kα X-ray source.

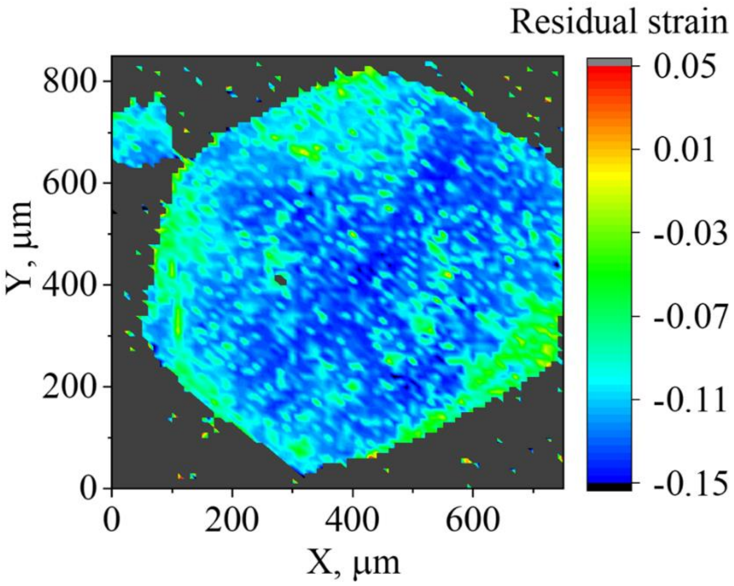

Supplementary Fig. 9:

Raman spectroscopy contour of a giant flake's residual strain.

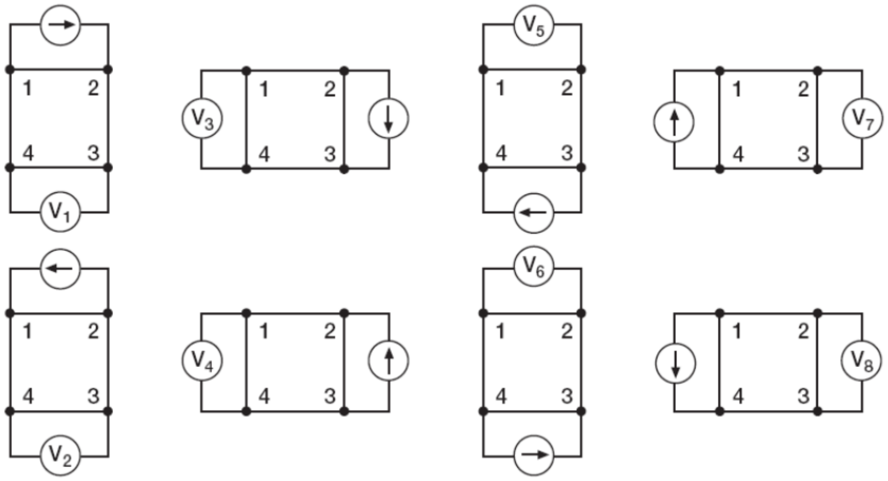

Supplementary Fig. 10:

Schematic of the van der Pauw configuration used in the determination of the sheet resistance.

Supplementary Movie Captions:

Supplementary Movie 1.

This movie illustrates a typical 'pulsed' growth leading to an assembly of hexagonal flakes with a hexagonal order, as presented in Fig 1A–E. The time of each movie frame is inserted, as well as the evolution of hydrogen over methane pressure ratio and a 100 μm scale bar. See Fig. 1A–E and Supplementary Movie 1.

Supplementary Movie 2.

This movie illustrates how the growth can be manipulated by successive sequences of growth and etching (achieved by decreasing the methane pressure over hydrogen pressure ratio), as presented in Fig. 2A–E. The time of each image, the CH_4 over H_2 pressure ratio and a 500 μm scale bar are inserted. See Fig. 2A–E and Supplementary Movie 2.

Supplementary Movie 3.

This movie corresponds to the growth of a single large flake as discussed in Fig. 3A–E. The time of each image, the H_2 over CH_4 pressure ratio and a 100 μm scale bar are inserted. See Fig. 3 and Supplementary Movie 3.

References:

(1) Lu, W.; Zeng, M.; Li, X.; Wang, J.; Tan, L.; Shao, M.; Han, J.; Wang, S.; Yue, S.; Zhang, T.; Hu, X.; Mendes, R. G.; Rümmeli, M. H.; Peng, L.; Liu, Z.; Fu, L. Controllable Sliding Transfer of Wafer-Size Graphene. *Adv. Sci.* **2016**, *3* (9), 1600006. https://doi.org/10.1002/advs.201600006.

(2) Lee, G. H. High-Strength Chemical-Vapor–Deposited Graphene and Grain Boundaries. *Science* **2013**, *340*, 1073–1076.

(3) Zhao, H.; Alurua, N. R. Temperature and Strain-Rate Dependent Fracture Strength of Graphene. *J Appl Phys* **2010**, *108*, 064321.

(4) Munson, R. A. Surface Energies of Liquid Metal Interfaces with Carbon. *Carbon* **1967**, *5*, 471.

(5) Khare, R. Coupled Quantum Mechanical/Molecular Mechanical Modeling of the Fracture of Defective Carbon Nanotubes and Graphene Sheets. *Phys B* **2007**, *75*, 075412.

(6) Zhang, P. Fracture Toughness of Graphene. *Nat. Commun* **2014**, *5*, 3782.

(7) Terasawa, T.; Saiki, K. Radiation-Mode Optical Microscopy on the Growth of Graphene. *Nat. Commun.* **2015**, *6*, 6834. https://doi.org/10.1038/ncomms7834.

(8) Nagata, K.; Nagane, T.; Susa, M. Measurement of Normal Spectral Emissivity of Liquid Copper. *ISIJ Int.* **1997**, *37* (4), 399–403. https://doi.org/10.2355/isijinternational.37.399.

(9) Freitag, M.; Chiu, H.-Y.; Steiner, M.; Perebeinos, V.; Avouris, P. Thermal Infrared Emission from Biased Graphene. *Nat. Nanotechnol.* **2010**, *5* (7), 497–501. https://doi.org/10.1038/nnano.2010.90.

(10) Muley, S. V.; Ravindra, N. M. Emissivity of Electronic Materials, Coatings, and Structures. *JOM* **2014**, *66* (4), 616–636. https://doi.org/10.1007/s11837-014-0940-0.

(11) Kalbac, M.; Frank, O.; Kavan, L. The Control of Graphene Double-Layer Formation in Copper-Catalyzed Chemical Vapor Deposition. *Carbon* **2012**, *50* (10), 3682–3687. https://doi.org/10.1016/j.carbon.2012.03.041.

(12) Nemes-Incze, P.; Yoo, K. J.; Tapasztó, L.; Dobrik, G.; Lábár, J.; Horváth, Z. E.; Hwang, C.; Biró, L. P. Revealing the Grain Structure of Graphene Grown by Chemical Vapor Deposition. *Appl. Phys. Lett.* **2011**, *99* (2), 023104. https://doi.org/10.1063/1.3610941.

(13) Zhang, Y.; Li, Z.; Kim, P.; Zhang, L.; Zhou, C. Anisotropic Hydrogen Etching of Chemical Vapor Deposited Graphene. *ACS Nano* **2012**, *6* (1), 126–132. https://doi.org/10.1021/nn202996r.

(14) Danov, K. D.; Kralchevsky, P. A.; Naydenov, B. N.; Brenn, G. Interactions between Particles with an Undulated Contact Line at a Fluid Interface: Capillary Multipoles of Arbitrary Order. *J. Colloid Interface Sci.* **2005**, *287* (1), 121–134. https://doi.org/10.1016/j.jcis.2005.01.079.

(15) Chan, D. Y. C.; Henry, J. D., Jr.; White, L. R. The Interaction of Colloidal Particles Collected at Fluid Interfaces. *J Colloid Interf Sci* **1981**, *79*, 410–418.

(16) Danov, K. D.; Kralchevsky, P. A. Interaction between Like-Charged Particles at a Liquid Interface: Electrostatic Repulsion *vs.* Electrocapillary Attraction. *J Colloid Interf Sci* **2010**, *345*, 505–514.

(17) Danov, K. D.; Kralchevsky, P. A. Capillary Forces between Particles at a Liquid Interface: General Theoretical Approach and Interactions between Capillary Multipoles. *Adv. Colloid Interface Sci.* **2010**, *154* (1–2), 91–103. https://doi.org/10.1016/j.cis.2010.01.010.

(18) Assael, M. J.; Kalyva, A. E.; Antoniadis, K. D. Reference Data for the Density and Viscosity of Liquid Copper and Liquid Tin. *J Phys Chem Ref Data* **2010**, *39*, 033105.

(19) Matsumoto, T.; Fujii, H.; Ueda, T.; Kamai, M.; Nogi, K. Measurement of Surface Tension of Molten Copper Using the Free-Fall Oscillating Drop Method. *Meas Sci Technol* **2005**, *16*, 432–437.

(20) Paunov, V. N.; Kralchevsky, P. A.; Denkov, N. D.; Nagayama, K. Lateral Capillary Forces between Floating Submillimeter Particles. *J Colloid Interf Sci* **1993**, *157*, 100–112.

(21) Khomyakov, P. A. First-Principles Study of the Interaction and Charge Transfer between Graphene and Metals. *Phys Rev B* **2009**, *79*, 195425.

(22) Georgantzinos, S. K.; Giannopoulos, G. I.; Fatsis, A.; Vlachakis, N. V. Analytical Expressions for Electrostatics of Graphene Structures. *Phys. E* **2016**, *84*, 27–36.

(23) Andersen, M.; Cingolani, J. S.; Reuter, K. *Ab Initio* Thermodynamics of Hydrocarbons Relevant to Graphene Growth at Solid and Liquid Cu Surfaces. *J Phys Chem* **2019**, *C 123*, 22299–22310.

(24) Plimpton, S. Fast Parallel Algorithms for Short-Range Molecular-Dynamics. *J Comput Phys* **1995**, *117*, 1–19.

(25) Larsen, A. H. The Atomic Simulation Environment- A Python Library for Working with Atoms. *J Phys Condens Matter* **2017**, *29*, 273002.

(26) Liang, T.; Devine, B.; Phillpot, S. R.; Sinnott, S. B. Variable Charge Reactive Potential for Hydrocarbons to Simulate Organic-Copper Interactions. *J. Phys. Chem. A* **2012**, *116* (30), 7976–7991. https://doi.org/10.1021/jp212083t.

(27) Liang, T. Classical Atomistic Simulations of Surfaces and Heterogeneous Interfaces with the Charge-Optimized Many Body (COMB) Potentials. *Mater Sci Eng Rep* **2013**, *74*, 255–279.

(28) Perdew, J. P.; Burke, K.; Ernzerhof, M. Generalized Gradient Approximation Made Simple. *Phys Rev Lett* **1996**, *77*, 3865–3868.

(29) Andersen, M.; Hornekær, L.; Hammer, B. Graphene on Metal Surfaces and Its Hydrogen Adsorption: A Meta-GGA Functional Study. *Phys Rev B* **2012**, *86*, 085405.

(30) Olsen, T.; Yan, J.; Mortensen, J. J.; Thygesen, K. S. Dispersive and Covalent Interactions between Graphene and Metal Surfaces from the Random Phase Approximation. *Phys Rev Lett* **2011**, *107 156401*.

(31) Horozov, T. S.; Aveyard, R.; Clint, J. H.; Binks, B. P. Order−Disorder Transition in Monolayers of Modified Monodisperse Silica Particles at the Octane−Water Interface. *Langmuir* **2003**, *19*, 2822–2829.

(32) Boneva, M. P.; Danov, K. D.; Christov, N. C.; Kralchevsky, P. A. Attraction between Particles at a Liquid Interface Due to the Interplay of Gravity- and Electric-Field-Induced Interfacial Deformations. *Langmuir* **2009**, *25*, 9129–9139.
